# Marine Drugs from Sponge-Microbe Association—A Review

**DOI:** 10.3390/md8041417

**Published:** 2010-04-22

**Authors:** Tresa Remya A. Thomas, Devanand P. Kavlekar, Ponnapakkam A. LokaBharathi

**Affiliations:** Biological Oceanography, National Institute of Oceanography, Dona Paula, Goa, Pin-403004, India; E-Mails: remyat@nio.org (T.R.A.T.); devanand@nio.org (D.P.K.)

**Keywords:** marine drugs, sponges, microbial symbionts, bioactive compounds

## Abstract

The subject of this review is the biodiversity of marine sponges and associated microbes which have been reported to produce therapeutically important compounds, along with the contextual information on their geographic distribution. Class Demospongiae and the orders Halichondrida, Poecilosclerida and Dictyoceratida are the richest sources of these compounds. Among the microbial associates, members of the bacterial phylum Actinobacteria and fungal division Ascomycota have been identified to be the dominant producers of therapeutics. Though the number of bacterial associates outnumber the fungal associates, the documented potential of fungi to produce clinically active compounds is currently more important than that of bacteria. Interestingly, production of a few identical compounds by entirely different host-microbial associations has been detected in both terrestrial and marine environments. In the Demospongiae, microbial association is highly specific and so to the production of compounds. Besides, persistent production of bioactive compounds has also been encountered in highly specific host-symbiont associations. Though spatial and temporal variations are known to have a marked effect on the quality and quantity of bioactive compounds, only a few studies have covered these dimensions. The need to augment production of these compounds through tissue culture and mariculture has also been stressed. The reviewed database of these compounds is available at www.niobioinformatics.in/drug.php.

## 1. Introduction

Sponges (Phylum: Porifera) are evolutionarily ancient metazoans that have existed for 700–800 million years. They not only populate the tropical oceans in great abundance but also occur in temperate waters and even in freshwater [1,2]. Marine sponges are widely distributed from intertidal zones to thousands of meters deep in the ocean [3]. They are simple multicellular invertebrates attached to solid substrates in benthic habitats. Sponges are filter feeders, having numerous tiny pores on their surface, which allow water to enter and circulate through a series of canals where microorganisms and organic particles are filtered out and eaten [4]. There are mainly three classes of sponges, namely the Calcarea (five orders and 24 families), Demospongiae (15 orders and 92 families) and Hexactinellida (six orders and 20 families). So far about 15,000 species of sponges have been described, but their true diversity may be higher [5]. Most of them occur in the marine environment and only about 1% inhabit freshwater [6]. Most of the species are placed under the class Demospongiae. Since sponges are simple and sessile organisms; during evolution they have developed potent chemical defensive mechanism to protect themselves from competitors and predators as well as infectious microorganisms. Studies show that secondary metabolites in sponges play a crucial role in their survival in the marine ecosystem [7]. These natural products have interesting biomedical potential, pharmaceutical relevance and diverse biotechnological applications [4,8–13]. The biomedical and pharmaceutical importances of these compounds are attributed to their antiviral, antitumor, antimicrobial and general cytotoxic properties [14]. Interestingly, out of the 13 marine natural products that are currently under clinical trials as new drug candidates, 12 are derived from invertebrates. Among them, Porifera remains the most important phylum, as it provides a greater number of natural products, especially novel pharmacologically active compounds [15,16]. Biochemical characteristics seem to be useful taxonomic markers and good indicators of sponge phylogeny [17]. The diversity of biochemical properties of sponges has been demonstrated by the continued discovery of novel compounds, having pharmacological properties [18]. These investigations started with the pioneering work of Bergmann on the extraction of novel bioactive nucleosides from the sponge *Tectitethya crypta* (formerly *Cryptotethya crypta*) [19]. The chemical diversity of secondary metabolites isolated from sponges includes amino acids, nucleosides, macrolides, porphyrins, terpenoids, aliphatic cyclic peroxides and sterols [7]. Sponges are well known to be hosts for a large community of microorganisms, which comprise a significant percentage (up to 50–60%) of the biomass of the sponge host [20,21]. The role of these diverse microbes in sponge biology varies from source of nutrition to mutualistic symbiosis with the sponge [22]. Based on bacterial community studies employing molecular methods such as Denaturing Gradient Gel Electrophoresis (DGGE), 16S rRNA gene sequencing and Fluorescence *In Situ* Hybridization (FISH), it has been recognized that the sponge-associated bacterial community consists of at least ten bacterial phyla such as Proteobacteria, Nitrospira, Cyanobacteria, Bacteriodetes, Actinobacteria, Chloroflexi, Planctomycetes, Acidobacteria, Poribacteria and Verrucomicrobia besides members of the domain Archaea [1,23–30]. Other symbiotic microbial populations that inhabit sponges are fungi and microalgae. Little is known about viruses in sponges, although virus-like particles have been observed in cell nuclei of *Aplysina (Verongia) cavernicola* [32]. There are two pathways through which a developing sponge acquires bacterial symbionts. The first one is by selective absorption of specific bacteria from the large diversity of bacteria in the surrounding water column that passes through the sponge during filter feeding. The second one is by vertical transmission of symbionts through the gametes of the sponge by inclusion of the bacteria in the oocytes or larvae [33].

Symbiotic functions that have been attributed to microbial associates include nutrient acquisition, stabilization of sponge skeleton, processing of metabolic waste and secondary metabolite production [1]. It is hypothesized that symbiotic marine microorganism harboured by sponges are the original producers of these bioactive compounds [12,33–35]. The first experimental evidence supporting this hypothesis was derived from the work of Faulkner *et al.* [27], who investigated the localization of natural products within sponge-microorganism association. For this purpose, cell populations within sponge samples were separated by differential centrifugation and the fractions obtained were analyzed chemically. By this approach, it was possible to locate the cytotoxic macrolide swinholide A and the peptide theopalauamide in the heterotrophic unicellular bacteria and in the filamentous heterotrophic bacteria, respectively. Both the bacterial strains were isolated from the sponge *Theonella swinhoei*.

Microbial associates of sponges gained significance as source of bioactive compounds only when a remarkable similarity was found between those compounds isolated predominantly from sponges and those found in terrestrial organism of entirely different taxa [36]. Likewise, similarities between the structures of mycalamide A & B from the marine sponge *Mycale hentscheli*, collected in Dunedin Harbour (New Zealand) and pederin, a toxin originally isolated from the *Paederus* beetle in South America was recognized by Perry *et al*. [37]. Mycalamides have been reported to be potent inhibitors of protein synthesis and were recently found to cause apoptosis [38]. Thus, it indicates that at least some of the bioactive secondary metabolites isolated from sponges are produced by functional enzyme clusters, originated from the sponges and/or their associated microorganisms [39]. It is now known that polybrominated biphenyl ether antibiotics isolated from the sponge *Dysidea herbacea* (Demospongiae) are actually produced by the endosymbiotic cyanobacterium *Oscillatoria spongeliae* [35,40]. Molecular methods (e.g; rDNA, DGGE and FISH) have revealed the association of a variety of unculturable bacteria and Archaea in sponges. It has recently been demonstrated that sponge isolates with antimicrobial activity are numerically very abundant in the genus *Pseudoalteromonas* and the group of α-Proteobacteria [7,41] and Actinobacteria [42]. As infectious microorganisms evolve and develop resistance to existing pharmaceuticals, marine sponges provide novel leads against bacterial, viral, fungal and parasitic diseases [39]. Thus, it is extremely relevant to highlight the therapeutic properties of various secondary metabolites synthesized by the microbial flora inhabiting sponges. In this review, an effort has been made to relate the biomedical significance of secondary metabolites of sponge-microbial association, which were discovered so far and their richness in different sponge taxa. It is also important to understand their ecological distribution in space and time so as to enable harnessing these compounds in an optimal and sustainable manner.

No bioactive compounds have been reported from microbes associated with sponge families such as Agelasidae, Astroscleridae, Calthropellidae, Geodiidae, Pachastrellidae, Thrombidae, Dictyodendrillidae, Acanthochaetetidae, Alectonidae, Hemiasterellidae, Placospongiidae, Polymastiidae, Spirastrellidae, Stylocordylidae, Tethyidae, Timeidae, Trachycladidae, Bubaridae, Dictyonellidae, Heteroxyidae, Halisarcidae, Calcifibrospongiidae, Phloeodictyidae, Lubomirskiidae, Malawispongiidae, Metaniidae, Metschnikowiidae, Palaeospongillidae, Potamolepiidae, Spongillidae, Spongillina incertae sedis, Plakinidae, Azoricidae, Corallistidae, Desmanthidae, Isoraphiniidae, Lithistida incertae sedis, Macandrewiidae, Phymaraphiniidae, Phymatellidae, Pleromidae, Scleritodermidae, Siphonidiidae, Vetulinidae, Latrunculiidae, Microcionidae, Rhabderemiidae, Cladorhizidae, Desmacellidae, Esperiopsidae, Guitarridae, Hamacanthidae, Merliidae, Podospongiidae, Chondropsidae, Coelosphaeridae, Crambeidae, Crellidae, Dendoricellidae, Desmacididae, Hymedesmiidae, Iotrochotidae, Phellodermidae, Tedaniidae, Samidae and Spirasigmidae of the class Demospongiae; Baeriidae, Lepidoleuconidae, Trichogypsiidae, Achramorphidae, Amphoriscidae, Grantiidae, Heteropiidae, Jenkinidae, Lelapiidae, Leucosoleniidae, Sycanthidae, Sycettidae, Minchinellidae, Petrobionidae, Clathrinida incertae sedis, Clathrinidae, Leucaltidae, Leucascidae, Levinellidae, Soleneiscidae, Lelapiellidae, Murrayonidae, Paramurrayonidae of the class Calcarea. There are no reports of microbially originated bioactive compounds from the class Hexactinellida.

## 2. Sponges and Associated Microbes Involved in Drug Production

### 2.1. Class: Demospongiae

#### 2.1.1. Order: Astrophorida

Family: Ancorinidae

l,l-Diketopiperazine known as cyclo-(l-Pro-l-Phe), showing moderate antimicrobial activity was isolated from the bacterium *Alcaligenes faecalis* A72, which was found in association with the South China Sea sponge *Stelletta tenuis* [42]. The sponge *Stelletta tenuis* is known for harbouring large number of cultivable bacterial diversity, including α-, γ-, δ-Proteobacteria, Bacteroidetes, Firmicutes and Actinobacteria [23,42]. A marine fungus of the class Hyphomycetes was isolated from the Indo-Pacific sponge *Jaspis aff. johnstoni*. Fermentation of this marine culture led to the isolation of the tricyclic sesquiterpenes coriolin B, dihydrocoriolin C as well as the novel chloriolines A-C. Coriolin B and dihydrocoriolin C were earlier isolated from the terrestrial wood-rotting basidiomycete *Coriolus consors*. Coriolin B exhibited strong inhibition of human breast and CNS cell lines with IC_50_ values of 0.7 μg (breast) and 0.5 μg (neuroblastoma) [9,43–45].

#### 2.1.2. Order: Chondrosida

Family: Chondrillidae

Seven new fungal polyketides were isolated from the mycelium extract of the fungus *Penicillium rugulosum*, derived from the sponge *Chondrosia reniformis* (Elba, Italy). They include prugosenes A1–A3, B1, B2, C1 and C2. These compounds can be used as templates for new anti-infectives [46–48].

#### 2.1.3. Order: Dendroceratida

Family: Darwinellidae

The sponge *Dendrilla nigra* is a rich source of cultivable marine actinomycetes. Investigations on a sponge specimen collected from the Vizhinjam coast (west coast of India) revealed that *Micromonospora-Saccharomonospora-Streptomyces* group was the major cultivable actinobacteria found in the sponge [49]. The species *Streptomyces dendra* sp. nov. MSI051 isolated from *Dendrilla nigra* from the same coast exhibited a broad spectrum of antibacterial activity. The host sponge, as well as the associated bacterial symbiont MSI051, contained high levels of PLA2 (Phospholipase A2) [50]. Since PLA2 is a well-established antibacterial protein in the defense system of higher animals, its presence in the sponge-associated bacteria may indicate an integrated functional role in the host defense system [51]. Another strain, *Streptomyces* sp. BLT7 isolated from *Dendrilla nigra* obtained from Kanyakumari (south east coast of India) also showed potential antibacterial activity in their extracellular products [52,53]. A number of actinobacterial strains were also obtained from *Dendrilla nigra*, collected from the southwest coast of India. Among eleven heterotrophic actinobacteria isolated from one specimen, *Nocardiopsis dassonvillei* MAD08 was prominent in its antibacterial and anticandidal activity against the multidrug resistant pathogenic microbial strains. The antibacterial activity was assigned to the presence of 11 compounds and the anticandidal activity to a single protein. The uniqueness of this strain is reflected in the expression of both organic solvent (antibacterial) and water soluble (antifungal) antimicrobial compounds. In future, this may lead the way towards large-scale profitable production of antimicrobials from *Nocardiopsis dassonvillei* MAD08 [53]. The above studies reflect the consistent production of antimicrobial compounds by the actinobacteria harbouring individuals of *Dendrilla nigra* from south west coast of India.

#### 2.1.4. Order: Dictyoceratida

##### 2.1.4.1. Family: Dysideidae

Many marine sponges, especially the tropical ones, form symbioses with algae and often become net primary producers. Although associations with cyanobacteria are the most common, such partnership has also been observed with chlorophytes, rhodophytes, dinoflagellates and diatoms [54,55]. A variety of marine sponges hold cyanobacteria as autotrophic symbionts, which are known to contribute to nutrition of host through extracellular lysis and phagocytosis, with possible glycogen reutilization by sponge cells. Cyanobacteria transfer glycerol and organic phosphate to sponge tissue, as derivatives of these compounds are known to support several basic metabolic pathways. Moreover, symbiotic cyanobacteria appear to be capable of fixing nitrogen [55]. The tropical marine shallow water sponge *Lamellodysidea herbacea* (formerly *Dysidea herbacea*) which is common throughout the Indo-Pacific, is always found to harbour filamentous non-heterocystous cyanobacterium *Oscillatoria spongeliae*. It occurs intercellularly in large numbers up to 20% of the symbiotic associations’ volume and 30–50% of the sponge tissue volume [10,57]. These cyanobacterial symbionts have been reported to be responsible for the production of a wide array of secondary metabolites by the sponge [55]. Nuclear magnetic resonance analysis of the symbiont cell preparations from the specimen of *Lamellodysidea herbacea* obtained from Great Barrier Reef, Australia showed that they usually contain the chlorinated diketopiperazines, dihydrodysamide C and didechlorodihydrodysamide C, which are characteristic metabolites of the sponge-symbiont association [56,57]. Since diketopiperazines (DKPs) are a common motif in various biologically active natural products, they may be useful scaffolds for the rational design of receptor probes and therapeutic agents [58]. Symbiotic microorganisms of *Dysidea* sp. can synthesize physiologically active compounds which belong to the group of brominated diphenyl ethers. *Vibrio* sp. associated with *Dysidea* specimen collected near the islands of Tutuila and Ofu (Eastern Samoa) synthesize cytotoxic and antibacterial tetrabromodiphenyl ethers [59]. A specimen of *Lamellodysidea herbacea* collected from the Republic of Palau (Caroline Island, Western Pacific Ocean) yielded a polybrominated biphenyl ether such as 2-(2′,4′-dibromophenyl)-4,6-dibromophenol. The compound was deposited as conspicuous crystals throughout the sponge tissue. The cyanobacteria *Oscillatoria spongeliae* was also observed as endosymbiont in the sponge mesohyl. They were separated from the sponge cells and heterotrophic bacteria by flow cytometry. Coupled gas chromatography-mass spectrometry and protein nuclear magnetic resonance revealed that the real source of the compound was the cyanobacteria *Oscillatoria spongeliae*. The polybrominated metabolites produced by the cyanobacteria are excreted into the surrounding aqueous medium in which they are not soluble, and therefore crystallize. Thus considerable amount of brominated metabolites are seen as crystalline material in the sponge mesohyl, with only a relatively small amount in the cyanobacteria. Polybrominated biphenyl ethers from *Lamellodysidea herbacea* are active against both Gram-, Gram+ bacteria and unicellular marine cyanobacteria. The compound, 2-(2′,4′-dibromophenyl)-4,6-dibromophenol showed antibacterial activity against *Staphylococcus aureus*, *Escherichia coli*, *Bacillus subtilis etc*. The apparent general toxicity of polybrominated compounds particularly to prokaryotes is beneficial to the association *Lamellodysidea herbacea*-*Oscillatoria spongeliae*. This association is more resistant to these compounds [40]. *Lamellodysidea herbacea* is one of the established model systems for addressing the question as to whether sponge metabolites are produced by the symbiotic bacterium or the host itself [60]. An unknown bacterium associated with the marine sponge *Dysidea avara*, collected from Adriatic Sea was found to produce the compound 2-methylthio-1,4-naphthoquinone. This compound showed strong antiangiogenic and antimicrobial properties [61]. 16S rDNA analysis revealed that the bacterial strain shares 99% identity to the α-Proteobacteria MBIC3368 [62].

##### 2.1.4.2. Family: Irciniidae

Marine sponges in the genus *Ircinia* are known to be good sources of secondary metabolites having biological activities [61,63,64]. The species *Ircinia fasciculata*, collected from the shallow coastal habitats of the Mediterranean Sea (~15 m depth) showed antimicrobial activity in the agar media inoculated with different indicator organisms such as *Escherichia coli*, *Staphylococcus lentus*, *Candida* sp., *Bacillus subtilis* and *Mycobacterium* sp. The sponge specimen was chosen for the isolation of bacteria, on the basis of the accumulated evidence that microorganism could well be the true source for some of the metabolites produced by sponges. γ-Proteobacteria was detected in the sponge isolate [32]. An antileukemic marine natural product, sorbicillactone A was isolated from the salt water culture of the fungus *Penicillium chrysogenum* obtained from another Mediterranean specimen of *Ircinia fasciculata.* It possesses a unique bicyclic lactone structure, seemingly derived from sorbicillin. The compound exhibited promising activities in several mammalian and viral test systems, particularly in a highly selective cytostatic activity against murine leukemic lymphoblasts (L5178y) and also showed the ability to protect human T cells against the cytopathic effects of HIV-1. These properties qualify sorbicillactone A for future therapeutic human trials [64,65].

##### 2.1.4.3. Family: Spongiidae

An antibacillus compound, which was chemically identified as the peptide antibiotic andrimid was detected in the extract of the sponge *Hyatella* sp. A bacterial isolate M22-1, belonging to the genus *Vibrio* was also isolated from the homogenate of the same sponge. The bacterium when cultured in marine agar also produced the same compound. This suggests that the origin of andrimid in the sponge is from the bacterium [66]. Andrimid previously isolated from the cultures of an *Enterobacter* sp. which is an intracellular symbiont of the brown plant hopper *Nilaparvata lugens* and was found to exhibit potent activity against *Xanthomonas campestris pv. oryzae* [67]. It has also been isolated from marine *Pseudomonas fluorescens*, which was active against methicillin-resistant *Staphylococcus aureus*. Due to the diversity of the microorganism producing this toxin, one can speculate that the production of this compound might be encoded by genes transferable on a plasmid [68]. The culture broth extracts of the fungus, *Myrothecium verrucaria* 973023 which was separated from *Spongia* sp. of Hawaii, showed potent activity against murine lymphocytic leukemia L1210 and human colon tumor H116 cell lines in the soft agar-based bioassay system. Further studies indicated the presence of three new trichothecenes, viz. 3-hydroxyroridin E, 13′-acetyltrichoverrin B, miophytocen C and nine known related compounds such as roridin A, L, M, isororidin A, epiroridin E, verrucarin A, M, trichoverrin A and B in the extract. All the compounds except miophytocen C showed significant cytotoxicity against murine and human tumor cell lines [69].

##### 2.1.4.4. Family: Thorectidae

A new antibiotic trisindole derivative, viz. trisindoline, has been characterized from a marine *Vibrio* sp., which was separated from the fresh marine okinawan sponge *Hyrtios altum*. Trisindoline was shown to exhibit potential antibiotic activity against *Escherichia coli*, *Bacillus subtilis* and *Staphylococcus aureus* [70–72]. An antileukemic compound, asperazine was isolated from the saltwater culture of the fungus *Aspergillus niger* obtained from a caribbean *Hyrtios* sponge by Crews *et al*. [73]. Asperazine is a member of a large family of diketopiperazine alkaloids. Asperazine displayed remarkable cytotoxicity and an interesting leukemia selectivity [45,74]. Culture extract of another strain of *Aspergillus niger* from the sponge *Hyrtios proteus* (Dry Tortugas National Park, Florida) displayed broad chemodiversity and five compounds belonging to a wide range of biosynthetic classes were isolated. Among them, malformin C and asperazine displayed tumor and leukemia selective bioactivity [75]. From the above findings, it can be deduced that *Aspergillus niger* associated with two different species of *Hyrtios* inhabiting different geographical locations is capable of producing asperazine. It also gives insight in to the adaptability of a particular microbial associate to a particular sponge genus. An epibiotic bacterial strain *Pseudoalteromonas maricaloris* KMM 636T, isolated from the Great Barrier Reef sponge *Fascaplysinopsis reticulata* was the source of two brominated chromopeptides such as bromoalterochromide A and bromoalterochromide A. They showed moderate cytotoxicity to the eggs of the sea urchin *Strongylocentrotus intermedius* [48,76].

#### 2.1.5. Order: Hadromerida

##### 2.1.5.1. Family: Spirastrellidae

A polyketide, 14,15-secocurvularin was isolated from the saltwater culture of an unidentified fungus obtained from an Indonesian encrusting sponge *Spirastrella vagabunda* [77]. It was described as being mildly antibiotic against *Bacillus subtilis* when compared to tetracycline [78].

##### 2.1.5.2. Family: Suberitidae

*Suberites domuncula* is yet another excellent source for the recovery of bacteria having bioactive potential. This sponge typically grows on snail shells and has a compact, smooth, waxy and colorful surface. Bacteria were isolated from the sponge surface as well as from the laboratory-developed primmorphs of *Suberites domuncula* collected from northern Adriatic Sea. Two bacteria isolated from the sponge surface were identified as α-Proteobacterium MBIC3368 by using 16S rDNA sequences [79]. This bacterium has also been isolated from several other sponges (e.g., *Rhopaloeides odorabile*, *Aplysina aerophoba*) regardless of their taxonomic identity, geographic location or natural product profile [30,40]. Another bacterial isolate from the sponge surface showed 98.8% species level similarity to *Idiomarina loihiensis* (Alteromonadaceae). The bacteria on primmorph represented unidentified novel species of *Pseudomonas* [79]. Bioactive extracts of α-Proteobacterial strains from the sponge surface as well as *Pseudomonas* sp. associated with primmorph exhibited antiangiogenic, antimicrobial, hemolytic and cytotoxic properties. These bacterial extracts were strongly active against multidrug-resistant clinical strains of *Staphylococcus aureus* and *Staphylococcus epidermidis*, isolated from hospital patients. Extracts from *Idiomarina* species also showed hemolytic activity [15].

#### 2.1.6. Order: Halichondrida

##### 2.1.6.1. Family: Axinellidae

A cyclic depsipeptide, majusculamide C has been isolated from the metabolites of the sponge *Ptilocaulis trachys* collected at the Enewetak Atoll (Marshall Island, Pacific Ocean). It was originally isolated from the toxic blue-green alga *Lyngbya majuscula* obtained from the same site. Majusculamide C exhibited antifungal activity against pathogens of commercially important plants. This discovery proved that accumulation of cyanobacteria in sponges is diet derived [81,82]. A symbiotic fungal strain *Myrothecium* sp. JS9 in the marine sponge *Axinella* sp. from South China Sea was found to be an efficient producer of most effective antifungal metabolites roridin A and D (macrocyclic trichothecenes). Biologically, this class of compounds was reported to possess antileukemic, antimalarial, antimicrobial, phytotoxic and cytotoxic properties [83]. Structurally unique steroids, isocyclocitrinols A and 22-acetylisocyclocitrinol A were isolated from the extract of a saltwater culture of sponge derived fungus *Penicillium citrinum*, separated from the sponge *Axinella* sp., collected in Papua New Guinea. Both the steroid compounds exhibited weak antibacterial activity against *Staphylococcus epidermidis* and *Enterococcus durans* [84]. The ethyl acetate extract of *Penicillium* sp., derived from the Mediterranean sponge *Axinella verrucosa*, yielded the known compound communesin B and its new congeners communesins C and D, and the known compounds oxaline, griseofulvin and dechlorogriseofulvin. Oxaline is an antiproliferative agent which inhibits microtubule protein/purified tubulin polymerization, resulting in arresting cell cycle at the M-phase [85]. Griseofulvin is a widely used antifungal agent for the treatment of superficial dermatomycoses [86]. In several bioassays performed on different leukemia cell lines, the communesins exhibited moderate antiproliferative activity [87]. From a static culture of the fungal strain *Aspergillus niger* isolated from the Mediterranean sponge *Axinella damicornis*, eight secondary metabolites belonging to four entirely different structural classes were obtained. Among these, the new compound 3,3′-bicoumarin (bicoumanigrin A) showed moderate cytotoxicity against human cancer cell lines *in vitro*. Another compound, aspernigrin B displayed a strong neuroprotective effect by significantly reducing the increase of intracellular calcium concentration in rat cortical neurons stimulated with glutamic acid or quisqualic acid [88]. A crude extract from a small-scale culture of the fungus *Acremonium* sp. 021172C cultured from an A*xinella* sp. collected from Milne Bay (Papua New Guinea) displayed potent cytotoxicity in a primary screening using leukemia and solid tumor murine and human cancer cell lines. This prompted the growth of a larger-scale culture of the fungus to facilitate the purification of potential therapeutic metabolites which resulted in four new related linear octapeptides, RHM1, 2, 3 and 4, and the known peptaibiotic efrapeptins E, F, G, new efrapeptins Eα and H, known cyclic N-methylated scytalidamides A and B. Efrapeptins displayed antibacterial activity and potent cytotoxicity against murine and human cancer cell lines. RHM1 and RHM2 showed only weak cytotoxicity against murine cancer cell lines but RHM1 exhibited antibacterial activity [89,90]. These studies further confirm the potentiality of fungal metabolites from marine environment.

##### 2.1.6.2. Family: Halichondriidae

The halichondrids form the most important members of demosponges. They are of particular interest because the composition of secondary metabolites is influenced by the presence of prokaryotic symbionts [91]. Sponges of the genus *Halichondria* such as *Halichondria okadai* and *Halichondria melanodocia* provide good examples for the importance of microalgal association in the production of natural compounds recovered from these invertebrates. Both species of *Halichondria* contain the protein phosphatase inhibitor okadaic acid [14]. It was first isolated from the sponge *Halichondria okadai*, but, later it was found out that a dinoflagellate *Prorocentrum lima* produced the inhibitor [17]. Two unidentified bacteria of the genera *Pseudomonas* and *Alteromonas* have been isolated from *Halichondria Okadai* homogenates. The *Pseudomonas* sp. KK10206C produced a novel C_50_-carotenoid, okadaxanthine. It turned out to be a potent singlet oxygen quencher and a well known source of okadaic acid [61,92,93]. *Alteromonas* sp. was responsible for the production of a well-known lactam alteramide A. The genus *Alteromonas* was found commonly associated with marine sponges that produce macrolactam and amide ester compounds with cytotoxic and antimicrobial properties. The tetracyclic alkaloid alteramide A exhibited cytotoxic activity against leukemia P-388, lymphoma L-1210 and epidermal carcinoma KB cells [93–95]. A fungal strain, *Trichoderma harzianum* OUPS-N115, isolated from the Japanese specimen of *Halichondria okadai* yielded novel cytotoxic compounds such as trichodenone A, B and C. They exhibited significant cytotoxicity against leukemia P388 cell line [79,96,97]. A Gram-bacterial strain *Rubritalea squalenifasciens* HOact23^T^ obtained from *Halichondria okadai* yielded potent red pigmented antioxidants acyl glycol-carotenoic acids such as diapolycopenedioic acid xylosyl esters A, B and C [30,48,98]. Another *Halichondria* species, *Halichondria panacea*, which occurs abundantly in the Adriatic Sea, North Sea and Baltic Sea, was colonized by bacteria in its mesohyl compartment. Moreover, different specimens of *Halichondria panacea* collected from all the three seas harboured bacteria of same genera and indicated the dominance of the genus *Rhodobacter*, suggesting the symbiotic relationship of these bacteria with the sponge. Evidence has been presented to support that growth of bacteria in *Halichondria panacea* is maintained by a lectin produced from eukaryotic host. The organic extracts prepared from the sponge samples displayed cytotoxicity against leukemia cells, which supports the possibility of toxic bacteria in the sponges [99]. Bacteria synthesizing neuroactive compounds were also isolated from *Halichondria panacea*. Two such bacterial species were identified from this sponge which displayed the highest identity to *Antarcticum vesiculatum* and *Psychroserpens burtonensis* [100]. An actinobacterium *Microbacterium* sp. isolated from the sponge *Halichondria panacea* (Adriatic coast, Croatia) produced four glycoglycerolipids and one diphosphatidylglycerol when grown on marine broth and artificial sea water. The glycoglycerolipid, 2 (1-*O*-acyl-3-[*R*-glucopyranosyl-(1–3)-(6-*O*-acyl-*R*-mannopyranosyl)]glycerol), showed positive results for antitumor activities in the initial studies [101]. Novel cytotoxic compounds, designated as gymnastatins A-H, Q and R, cytotoxic ergastanoids such as gymnasterone A, B, C and D, novel class of steroid dankasterones A and B, and dankastatins A and B were isolated from an ascomycete fungal strain *Gymnascella dankaliensis* OUPS-N134, derived from the sponge *Halichondria japonica*. Gymnastatins A, B, C, F, G, Q and R, dankastatins A and B exhibited potent cytotoxicity and growth inhibition in a P388 lymphocytic leukemia test system in cell culture. Gymnastatin Q was equally active against breast and human cancer cell lines [78,102–107]. Gymnasterones B, C and D, and dankasterone A showed significant cytotoxic activity in P388 lymphocytic leukemia test system in cell culture. Dankasterone A was also active against human cancer cell lines [79,104,108,109]. Again from *Halichondria Japonica*, a fungal strain *Phoma* sp. Q60596 was obtained, which gave rise to the new antifungal antibiotic, YM-202204. It exhibited potent antifungal activities against *Candida albicans*, *Cryptococcus neoformans* and *Aspergillus fumigatus* [110]. Novel antibiotics, YM-266183 and YM-266184, were found in the culture broth of *Bacillus cereus* QN03323, which was isolated from *Halichondria japonica*. They exhibited potent antibacterial activities against staphylococci and enterococci including multiple drug resistant strains, whereas they were inactive against Gram-bacteria [111–113]. The antifungal macrolid halichondramide from another *Halichondria* sp. showed resemblance to the compound scytophycin B, which was extracted earlier from the cyanobacterium *Scytonema pseudohofmanni*, and therefore halichondramide is speculated to be of microbial origin [114]. Halichondramide also showed *in vitro* antimalarial activity [115]. The marine bacterial strain *Bacillus pumilus* AAS3 isolated from the Mediterranean sponge *Acanthella acuta*, produced a diglucosyl-glycerolipid, GGL11. Lipase catalyzed modification of this native substance led to the deacylated parent compound GG11. Antitumor promoting studies showed that the diglucosyl-glycerol GG11 strongly inhibited the growth of the tumor cell lines HM02 and Hep G2. Thus, it indicates the potential inhibitory activity of the compound with carbohydrate/glycerol backbone [116]. Twenty nine marine bacterial strains were isolated from the sponge *Hymeniacidon perlevis* at Nanji Island (China Sea), and the antimicrobial screening showed that eight strains inhibited the growth of terrestrial microorganisms. Among them, the strain NJ6-3-1 with wide antimicrobial spectrum was identified as *Pseudoalteromonas piscida* based on its 16S rRNA sequence analysis. The major antimicrobial metabolite isolated from this bacterium was norhman [43,117]. Another specimen of *Hymeniacidon perlevis* from the intertidal zone of Fujiazhuang coastline (China) was identified to be a good source of large amount of culturable and active epi/endophytic fungal strains. Of the various fungal isolates obtained from *Hymeniacidon perlevis*, the extracts of epiphytic fungus *Fusarium oxysporum* DLFP2008005 exhibited effective antibacterial and antifungal activities against Gram+ *Staphylococcus epidermidis*, *Bacillus subtilis*, Gram**-***Pseudomonas fluorescens*, *Pseudomonas aeruginosa* and the yeast *Candida albicans*. Several terrestrial as well as marine *Fusarium* species have been reported to produce structurally diversified antimicrobial compounds. The potential of fungi of the genus *Fusarium* as producers of novel antibiotics is therefore quite evident [44].

#### 2.1.7. Order: Haplosclerida

##### 2.1.7.1. Family: Callyspongiidae

An antimicrobial fungal metabolite known as acetyl Sumiki’s acid was isolated from a seawater-based fermentation of the fungal isolate *Cladosporium herbarum*, obtained from the marine sponge *Callyspongia aerizusa* in Indonesia. Both Sumiki’s acid and its acetyl derivative showed activity against *Bacillus subtilis* and *Staphylococcus aureus* at 5 μg/disc [118]. The tropical sponge *Callyspongia vaginalis* from the Caribbean Sea, yielded a new tyrosine kinase inhibitor and the antimicrobial compound ulocladol together with the antifungal agent 1-hydroxy-6-methyl-8-(hydroxyl-methyl)xanthone. These compounds have been extracted from the culture of sponge-derived fungi *Ulocladium botrylis* 193A4 [119,120].

##### 2.1.7.2. Family: Chalinidae

The marine sponge genus *Haliclona* has been extensively examined, and at least 190 metabolites exhibiting anti-fouling, antimicrobial, antifungal, antimalarial and cytotoxic activities have been isolated [121]. A fungal strain isolated from the sponge *Haliclona valliculata* collected from Elba, Italy and identified as *Emericella variecolor* showed a remarkable diversity of secondary metabolites. However, strains of the fungus *Emericella variecolor* have been the source of a variety of natural products. The culture of *Emericella variecolor* isolated from *Haliclona valliculata* proved to be chemically prolific. Among various compounds isolated, the novel anthraquinone, evariquinone revealed a strong antiproliferative activity against KB (ATCC CCL17, human cervix carcinoma) and NCI-H460 (NCI 503473, non-small cell lung cancer) cells [122]. Associated with *Haliclona simulans* from the west coast of Ireland, 52 bacteria isolated belonged to the genera *Pseudoalteromonas*, *Pseudomonas*, *Halomonas*, *Psychrobacter*, *Marinobacter*, *Sulfitobacter*, *Pseudovibrio*, *Salegentibacter*, *Bacillus*, *Cytophaga*, *Rhodococcus* and *Streptomyces* [23]. These strains were found to be rich sources of biological activities with over 50% exhibiting antimicrobial activities. Twelve *Streptomyces* and one *Bacillus* strain were found to produce substance active against drug-resistant pathogenic bacteria. PKS (polyketide synthase) and NRPS (nonribosomal peptide synthetase) genes found in Actinobacteria, *Bacillus*, *Sulfitobacter* and *Pseudovibrio*, suggest a high potential for secondary metabolite production by these organisms. Detection of wide spectrum antibiotic activities from *Streptomyces* isolates SM2 and SM4 is another evidence to support that culturable sponge microbiota is an important source of biologically active compounds. The saltwater culture of an unidentified fungus obtained from the sponge *Haliclona* sp. was found to produce several new hirsutane sesquiterpenes such as hirsutanols A–C and *ent*-gloeosteretriol. Hirsutanols are biosynthetically related to several compounds reported from the terrestrial fungus *Coriolus consors.* Hirsutanol A and *ent*-gloeosteretriol exhibited mild antibiotic activity against *Bacillus subtilis* [123]. Potent bacterial strains from *Haliclona* sp. (Bandangan water, North Java Sea, Indonesia) exhibiting antibacterial activity against the pathogenic bacteria such as *Vibrio parahaemolyticus*, *Aeromonas hydrophila* and *Staphylococcus aureus* were identified using rep-PCR followed by the construction of dendrogram and subsequent DNA sequencing. The active strains showed closest similarity to *Vibrio parahaemolyticus*, *Pseudovibrio denitrificans*, *Pseudoalteromonas* sp., α-Proteobacterium and uncultured bacterium clone [2].

##### 2.1.7.3. Family: Niphatidae

The fungus *Curvularia lunata* isolated from the marine sponge *Niphates olemda* from Indonesia yielded two antibacterial anthraquinones such as lunatin and cytoskyrin A. Both of them were found to be active against *Staphylococcus aureus*, *Escherichia coli* and *Bacillus subtilis* [124,125].

##### 2.1.7.4. Family: Petrosiidae

Genus *Petrosia* has been recognized as a source of diverse metabolites [126,127]. *Petrosia ficiformis* is a common Mediterranean sponge living in hard substrata between 5 and 45 m depth. Its colour mainly due to symbiotic cyanobacteria, ranges from violet to brown according to the illumination of environment. *Petrosia ficiformis* hosts a variety of heterotrophic bacteria, most of which live together with cyanobacteria within specialized cells called bacteriocytes [128]. Antimicrobial activity in several epibiotic bacterial isolates from *Petrosia ficiformis* has been observed by Chelossi *et al*. [129]. Two of these were identified as *Rhodococcus* sp. and *Pseudomonas* sp. by partial 16S rRNA gene sequencing. A strain of *Penicillium brevicompactum* derived from the specimen of *Petrosia ficiformis* provided two new cyclopentadepsipeptides, petrosifungins A and B along with the known fungal metabolites brevianamide A, mycophenolic acid (a well known immunosuppressive agent) and asperphenamate. Since cyclodepsipeptides constitute new class of potential drugs, petrosifungins A and B, may serve as lead compounds for more pharmacologically potent and toxicologically safe derivatives [130,131]. A strain of *Aspergillus insuetus* obtained from the surface of *Petrosia ficiformis* yielded two new compounds, terretonins E and F. They are potent inhibitors of mammalian mitochondrial respiratory chain [132]. One of the most potent antibacterial activities was detected in the crude extracts of a bacterial strain *Micrococcus luteus* R-1588-10, isolated from the surface of the sponge *Xestospongia* sp. collected from off Noumea (New Caledonia, southwest Pacific). *Micrococcus luteus* is an ubiquitous Gram+ bacteria. Two antimicrobial compounds such as 2,4,4′-trichloro-2′-hydroxydiphenylether (triclosan) and acyl-1-(acyl-6′-mannobiosyl)-3-glycerol (lutoside) have been isolated from *Micrococcus luteus* [133]. Fungal isolates of *Penicillium cf. montanense* obtained from the sponge *Xestospongia exigua* from Bali Sea (Indonesia) has yielded three novel decalactone metabolites, xestodecalactones A, B, and C [134]. Among these, xestodecalactone B showed antifungal activity against *Candida albicans* [79]. An antibacterial compound, aspergillitine was also isolated from *Xestospongia exigua* in association with the fungus *Aspergillus versicolor*. It showed moderate antibacterial activity against *Bacillus subtilis* [124,135]. An anti-infective alkaloid manzamine A was successfully obtained from the culture of the actinobacterium *Micromonospora* sp. harbouring the deep water Indonesian sponge *Acanthostrongylophora* sp. [82]. Manzamine alkaloids were reported earlier from several unrelated and geographically separated sponges, which suggest the microbial origin for the biosynthesis of these compounds [13,136]. Manzamine A was initially described as an antitumor agent against mouse leukemia cells [137] and recently shown to possess antimalarial properties that inhibit *in vivo* the growth of the rodent malarial parasite *Plasmodium berghei* [138]. Large scale culture of the sponge derived *Micromonospora* sp. has since been achieved in 20-litre fermentations, maintaining the manzamine production [13]. The fungus *Aspergillus versicolor*, isolated from *Petrosia* sp. (Jeju Island, Korea) yielded three known polyketides such as decumbenones A, B and versiol, and the cytotoxic lipopeptide fellutamide C. The same polyketides have been also reported from soil associated fungus *Penicillium decumbens*. Decumbenone A is a good inhibitor of melanin [139–141].

#### 2.1.8. Order: Lithistida

##### 2.1.8.1. Family: Neopeltidae

Lithistid sponges are renowned among marine organisms for their ability to produce a diverse array of biologically active metabolites [142], including novel peptides characterized by a high proportion of D and/or *N*-methylated amino acids. The similarity between lithistid peptides and those from microorganisms leads to the speculation that lithistid peptides might arise from symbiotic microbes [143]. A Gram-strain, 1537–E7 was identified as new *Pseudomonas* species from the surface of the sponge *Homophymia* sp. collected from off Touho (New Caledonia). Among the five compounds isolated from this bacterium, compound **1** (2-undecyl-4-quinolone) was active against the malarial parasite *Plasmodium falciparum* and HIV-1. Compound **2** (2-undecen-1′-yl-4-quinolone) displayed mild toxicity and compound **4** (2-nonyl-4-hydroxyquinoline *N*-oxide) showed antimicrobial activity against *Staphylococcus aureus* as well as cytotoxicity [142].

##### 2.1.8.2. Family: Theonellidae

The marine sponge *Theonella swinhoei* from Palau contains a cytotoxic polyketide, swinholide A and the bicyclic glycopeptide antifungal compound theopalauamide [144]. Bacteria associated with this sponge include unicellular cyanobacteria, unicellular bacteria and filamentous bacteria. Swinholide A is likely to be a bacterial metabolite because this compound was associated with fractions from unicellular bacteria in *Theonella swinhoei* [145]. A single morphotype of a filamentous bacterium was present in a separate fraction that contained the antifungal compound theopalauamide [146]. Subsequent application of molecular approaches identified this filamentous bacterium as novel δ-Proteobacterium related to myxobacteria. According to 16S rDNA data, the filamentous strain is a previously unknown δ-Proteobacterium with close association to the myxococcales and designated as ‘*Candidatus Entotheonella palauensis*’ [26]. An antifungal glycopeptide known as theonegramide was previously isolated from *Theonella swinhoei*, collected from Philippines at a depth of 20 m [147]. Interestingly, 16S sequences which showed 98% identity to that of the filamentous δ-Proteobacterium, *Entotheonella palauensis* were detected in *Theonella swinhoei* specimens containing the closely related metabolites theonegramide (from the Philippines) and theonellamide F (from Japan), while they were absent in sponges with different metabolites [148]. Theopalauamide-type compounds therefore, seem to be chemical markers for symbiosis of *Entotheonella palauensis* in sponges [145]. Discovery of *onn* genes encoding the biosynthesis of onnamide A in the microbial metagenome of the sponge *Theonella swinhoei* was made by Piel *et al.* [149]. This polyketide exhibited extremely potent antitumor activities. This provides the first experimental proof for bacterial origin of marine sponge derived natural compounds [150].

#### 2.1.9. Order: Poecilosclerida

##### 2.1.9.1. Family: Acarnidae

Three novel cytotoxic polyketides, brocaenols A-C were produced by *Penicillium brocae* obtained from a tissue sample of the Fijian sponge *Zyzzya* sp. When tested against HCT-116 cell line, all three compounds showed cytotoxicity [151,152].

##### 2.1.9.2. Family: Isodictyidae

An antibacterial compound known as cyclo*-*(l-proline-l-methionine) has been isolated from the culture broth of a symbiotic bacterium *Pseudomonas aeruginosa*, obtained from the Antarctic sponge *Isodictya setifera.* It showed antimicrobial activity against *Bacillus subtilis*, *Staphylococcus aureus*, *and Micrococcus luteus* [153].

##### 2.1.9.3. Family: Raspailiidae

A fungal strain *Coniothyrium* sp. 193477, isolated from the sponge *Ectyoplasia ferox* from the waters around the Caribbean Islands of Dominica, yielded novel antimicrobial compounds such as (3*S*)-(3′,5′-dihydroxyphenyl)butan-2-one and 2-(1′(*E*)-propenyl)-octa-4(*E*),6(*Z*)-diene-1,2-diol together with known fungal metabolites such as (3*R*)-6-methoxymellein, (3*R*)-6-methoxy-7-chloromellein and cryptosporiopsinol. Among these, cryptosporiopsinol demonstrated significant antimicrobial activity [154]. Potent cytotoxic compounds, epoxyphomalin A and B were discovered from *Phoma* sp., associated with *Ectyoplasia ferox* collected from the same region. The former one showed superior activity against various human tumor cell lines [155]. Another fungus *Spicellum roseum* 193H15, derived from *Ectyoplasia ferox* was found to produce trichothecenes such as trichodermol and 8-deoxytrichothecin. They considerably inhibited the activity of LacCer synthase (role in oncogene expression and cell proliferation) in neuroblastoma cells [156,157]. The fungus also yielded two cyclohexadepsipeptides, spicellamides A and B [48,158].

##### 2.1.9.4. Family: Mycalidae

An actinobacterium strain *Saccharopolyspora* sp. nov. associated with the sponge *Mycale plumose* from Qingdao coast (China) showed cytotoxic activities against temperature sensitive mutant cell lines of mouse (tsFT210). This led to the isolation of two prodigiosins analogs-metacycloprodigiosin and undecylprodigiosin. Prodigiosins are a family of naturally occurring polypyrrole red pigments produced by a restricted group of microorganisms including *Streptomyces* and *Serratia* strains. They are known to exhibit a wide range of biological activities. Both the above mentioned prodigiosin analogs exhibited potent *in vitro* cytotoxic activity against cancer cell lines such as P388, HL60, A-549, BEL-7402 and SPCA4 [159]. The fungus *Penicillium auratiogriseum* was also isolated from the specimen of *Mycale plumose* taken from the same geographical area. A new cytotoxic compound (*S*)-2,4-dihydroxy-1-butyl-(4-hydroxy) benzoate and a known compound fructigenine A were obtained from the fungus. Both the compounds were tested for their antitumor activity and exhibited potent cytotoxic effects [160]. Besides these, two new quinazoline alkaloids such as aurantiomides B and C showing moderate cytotoxic activities were isolated from another strain of *Penicillium auratiogriseum* associated with *Mycale plumose* from China [161]. Exophilin A, a new antibacterial compound, was discovered in the culture of the fungus *Exophiala pisciphila* NI10102, that was isolated from a marine sponge *Mycale adhaerens*. Exophilin A showed antimicrobial activity against Gram+ bacteria [48,162].

##### 2.1.9.5. Family: Myxillidae

A new antimicrobial fungal metabolite known as microsphaeropsisin together with the known compounds (*R*)-mellein, (3*R*,4*S*)-hydroxymellein, (3*R*,4*R*)-hydroxymellein and 4,8-dihydroxy-3,4-dihydro-2*H*-naphthalen-1-one were obtained from the fungal strain *Microsphaeropsis* sp. H5-50 associated with the marine sponge *Myxilla incrustance*, collected from Helgoland, Germany [154]. Microsphaeropsin, an eremophilane derivative showed antifungal activity at the 50 μg level [79].

#### 2.1.10. Order: Spirophorida

##### 2.1.10.1. Family: Tetillidae

A chitinase exhibiting antifungal activity was isolated from marine *Streptomyces* sp. DA11 associated with south China sponge *Craniella australiensis*. Compared with chitinase derived from terrestrial organisms, marine chitinase with higher pH and salinity tolerance may contribute to special biotechnological applications. Therefore, novel marine chitinase could be of great importance [163].

#### 2.1.11. Order: Verongida

##### 2.1.11.1. Family: Aplysinellidae

Ten strains of marine actinobacteria belonging to the genus *Salinospora* were isolated from the Great Barrier Reef sponge *Suberea clavata* (formerly *Pseudoceratina clavata*) [164]. The *Salinospora* group, a relatively newly discovered group of actinobacteria, has great applied potential. The *Salinospora* strains previously isolated from marine sediments showed significant cancer cell cytotoxicities as well as antifungal and antibiotic activities [165]. Significantly, *Salinospora* forms a potential new source of rifamycins and polyketide synthesis gene clusters specific to rifamycin synthesis. *Salinospora* isolate from *Suberea clavata* was found to produce compounds of the rifamycin class, including rifamycin B and rifamycin SV [166]. Other culturable symbiotic bacterial communities isolated from *Suberea clavata* include α-, γ-Proteobacteria, Bacteriodetes and Firmicutes [167].

##### 2.1.11.2. Family: Aplysinidae

Sponges of the Aplysinidae family are abundant in the subtropical and tropical waters of the Mediterranean Sea, Pacific and Atlantic Oceans [168]. *Aplysina* sponges harbour large amounts of microorganisms with antimicrobial activities that are embedded within the mesohyl [41,168]. The Mediterranean sponge *Aplysina aerophoba* is especially rich in bacteria. The amount of bacteria present in the sponge tissue matrix exceeds the microbial concentration of the seawater by two to three orders of magnitude [169]. One of the studies conducted using FISH on *Aplysina aerophoba* and its sibling species *Aplysina cavernicola* showed that the bacterial profiles of both species was very similar. Up to 40% of the sponge biomass consisted of bacteria and cyanobacteria. A large fraction of the microbial community was specific to and permanently associated with the host sponge [41,168,169]. The similarity of the bacterial communities in *Aplysina aerophoba* and *Aplysina cavernicola* corresponds to similarities in the natural product profiles of both sponges which are characterized by brominated alkaloids with cytotoxic activities and repellent properties against predators [12,41]. Among the bacterial isolates obtained from these species those which showed antimicrobial activity were numerically the most abundant in the genus *Pseudoalteromonas* and the class α-Proteobacteria. A general pattern was observed in that Gram+ bacteria inhibited Gram+ strains while Gram-bacteria inhibited Gram− isolates. Antimicrobial activities were also found against clinical isolates, *i.e.*, multi-drug resistant *Staphylococcus aureus* and *Staphylococcus epidermidis* strains isolated from hospital patients. The high recovery of strains with antimicrobial activity suggests that marine sponges represent an ecological niche which harbours largely uncharacterized microbial diversity and yet undiscovered metabolic potential [41]. Antimicrobial activity of bacterial isolates from *Aplysina aerophoba* collected from the Mediterranean coast of France has been tested against a set of standard Gram+, Gram− and eukaryotic microorganisms. The results showed that *Bacillus subtilis* strains A184, A190 and A202 exhibited strong activity against the fungus *Candida albicans* [170]. It is generally accepted that a combination of fungicidal and hemolytic activity in *Bacillus* is a valid indicator for the presence of lipopeptide from the iturin or surfactin class [171–173]. For the *Bacillus subtilis* strains A184, A190 and A202, these features are consistent. The results of MALDI MS which was applied to study the production of secondary metabolites by *Bacillus* species showed that strain A184 produced surfactins, iturins and fengycins while strain A190 produced surfactin and strain A202 produced iturin. The highly versatile strain *Bacillus subtilis* A184 was highly active against the multidrug resistant pathogenic *Staphylococcus aureus* and *Staphylococcus epidermidis*. Another species, *Bacillus pumilus* A586 demostrated high activity against *Staphylococcus aureus* and produced plumilacidin containing β-hydroxy fatty acid (surfactin like compound) [170]. An undescribed fungus of the genus *Microsphaeropsis*, isolated from the Mediterranean specimen of *Aplysina aerophoba*, was shown to produce a Protein Kinase C inhibitor known as 10-Hydroxy-18-methoxylbetaenone [174]. Since PKC plays an important role in neoplastic transformation, carcinogenesis and tumour cell invasion, those agents which inhibit the action of PKC are therapeutically very important [175].

##### 2.1.11.3. Family: Pseudoceratinidae

Extract of *Metarrhizium* sp. 001103 from *Pseudoceratina purpurea* (Fiji), yielded six known *N*-methylated cyclic depsipeptides of the destruxin family. They include destruxins A, B, B2, desmethyl B, E and E2 chlorohydrin. Destruxins A, B2, desmethyl B and E chlorohydrin displayed selective inhibition of human tumor cell lines. E2 chlorohydrin showed cytotoxicity towards murine c38 cell line. E chlorohydrin was the most potent among the group [90].

### 2.2. Class: Calcarea

Order: Clathrinida

Family: Leucettidae

Nonribosomal cyclic peptide leucamide A was isolated from the sponge *Leucetta microraphis*, obtained from the Great Barrier Reef of Australia. The compound was found to inhibit the growth of three tumor cell lines (stomach carcinoma, liver carcinoma and liver carcinoma with mutated p53). Leucamide A closely resembles the compound albeit, which is found frequently in cyanobacteria. Scanning electromicrographs of *Leucetta microraphis* revealed the presence of microbial symbionts, including cyanobacteria in the tissue. The sponge-derived leucamide A might, therefore be produced by cyanobacteria associated with it and not by the invertebrate itself [120].

### 2.3. Unidentified sponges

A marine-derived strain of the fungus *Emericella variecolor*, obtained from a Venezuelan sponge, yielded new compounds along with a group of known metabolites. Some of the novel compounds such as varitriol and varixanthone exhibited potent pharmaceutical activities. Varitriol displayed increased potency towards selected renal, CNS and breast cancer cell lines, whereas varixanthone showed antimicrobial activity [176]. Two novel antimycin antibiotics viz. urauchimycins A and B, were isolated from a fermentation broth of *Streptomyces* sp. Ni-80. The strain was isolated from an unidentified sponge. They are the first antimycin antibiotics which possess a branched side chain moiety. They exhibited inhibitory activity against morphological differentiation of *Candida albicans* [177]. A strain of the fungus *Microascus longirostris* SF-73 from a marine sponge collected at Harrington Point (Otago Harbour, New Zealand) was found to produce secondary metabolites such as cathestatin A, B and C, which strongly inhibited cystein proteases. Since specific and selective protease inhibitors are potentially powerful tools in clinical therapy, these inhibitors could be used in inactivating the target proteases in the pathogenic processes of human diseases such as emphysema, arthritis, pancreatitis, thrombosis, high blood pressure, muscular dystrophy, cancers, AIDS and many others [178]. Three antibacterial compounds were isolated from the fungus *Aspergillus ostianus* 01F313, derived from an unidentified sponge collected at Pohnpei (The federated state of Micronesia). They include 8-chloro-9-hydroxy-8,9-deoxyasperlactone, 9-chloro-8-hydroxy-8,9-deoxyasperlactone and 9-chloro-8-hydroxy-8,9-deoxyaspyrone [179]. From the same strain, five cytotoxic compounds such as aspinorene, dihydroaspyrone, aspergillides A, B and C were also obtained when cultured in a brominated medium. They exhibited cytotoxicity against lymphocytic leukemia cells (L1210) [48,180,181]. Cultivation of the fungus *Cryptosphaeria eunomia*, obtained from an unidentified sponge at Pohnpei yielded the antimycobacterial compounds diaporthein A and B. These compounds have been previously isolated from the terrestrial fungus *Diaporthe* sp. BCC 6140 [48,182,183].

## 3. Discussion

Sponge-microbial associations which synthesize clinically significant bioactive compounds have been discovered so far from geographically different regions such as Great Barrier Reef of Australia, South China Sea, Mediterranean Sea, Indonesia, Papua New Guinea, Indo-Pacific region *etc*. (Table 2). The review brings out the fact that members of the class Demospongiae are the richest producer of pharmacologically significant bioactive compounds in association with microbes. Out of 92 families under class Demospongiae, 26 familes have been identified to produce medicinally important bioactive compounds of microbial origin. They includes Ancorinidae, Chondrillidae, Darwinellidae, Dysideidae, Irciniidae, Spongiidae, Thorectidae, Spirastrellidae, Suberitidae, Axinellidae, Halichondriidae, Callyspongiidae, Chalinidae, Niphatidae, Petrosiidae, Neopeltidae, Theonellidae, Acarnidae, Raspailiidae, Isodictyidae, Mycalidae, Myxillidae, Tetillidae, Aplysinellidae, Aplysinidae and Pseudoceratinidae. The major orders which contribute maximum to the compound production are Halichondrida, Dictyoceratida and Poecilosclerida. Families which belong to the order Halichondrida such as Axinellidae and Halichondriidae are more influenced by microbes in the production of secondary metabolites. The microbial associates of halichondrid comprises broad spectrum of bacteria, actinobacteria, fungi and micro algae. Association of these microbes with different species of halichondrid sponges have been shown to be the real source of bioactive compounds exhibiting significant therapeutic effects. These compounds include alteramide, trichodenone A-C, gymnastatins A-C (antileukemic) YM-202204, YM-266183 and YM-266184 (antibiotics). Apart from these, species belonging to the families Chalinidae and Petrosiidae of the order Haplosclerida, Darwinellidae of the order Dendroceratida are also rich sources of bioactive compounds of microbial origin. Only one family from the class Calcarea has been identified as a source of pharmacologically significant bioactive compounds of microbial origin. There are no reports in the literature regarding isolation of microbial originated therapeutic compounds from the class Hexactinellida.

Some of the compounds produced by microbes in association with sponge orders such as Hadromerida, Haplosclerida and Verongida have not been characterized and therefore have not been included in the above figure.

The major groups of microorganisms recognized from this review as possible contributors of pharmacologically relevant secondary metabolites of sponges includes α, β, γ, δ- Proteobacteria, Firmicutes, Actinobacteria, Cyanobacteria and Fungi. Interestingly, the members of the fungal genus *Aspergillus*, which is ubiquitous in terrestrial, is also the principle source of bioactive compounds in marine sponges. Out of more than 680 fungal strains isolated worldwide from 16 sponge species, majority belong to the genera *Aspergillus* and *Penicillium* [184].The *Fusarium* genus is also considered as a potential candidate for the production of novel antibiotics [44]. Even though most of the the sponge-microbial association is very specific for the production of a particular compound, a few compounds have also been isolated from free living and associated microbes in marine and terrestrial ecosystems. Tricyclic sesquiterpene coriolin B (anticancer) has been isolated from a marine fungus of the class Hyphomycetes in *Jaspis. aff. johnstoni* as well as from the terrestrial wood rotting basidiomycete *Coriolus consors* [45]. Both the antimycobacterial compounds diaporthein A and B were isolated from the terrestrial fungus *Diaporthe* sp. and the marine fungus *Cryptosphaeria eunomia* associated with an unidentified sponge [48,182,183]. Polyketides such as decumbenones A and B were earlier isolated from the soil fungus *Penicillium decumbens* and later from *Aspergillus versicolor* associated with *Petrosia* sp. [139–141]. The antibacillus peptide antibiotic, associated with *Petrosia* sp. [139–141]. The antibacillus peptide antibiotic, andrimid was isolated from *Vibrio* sp. M 22-1 associated with the sponge *Hyatella* sp. and also from a symbiotic *Enterobacter* sp. of the brown plant-hopper *Nilaparvata lugens* [68]. Some sponges always harbour a particular genera or species of microorganism and consistently produce specific group of compounds. The association of the tropical marine shallow water sponge *Lamellodysidea herbacea* with cyanobacterium *Oscillatoria spongeliae*, is one such example which produces chlorinated diketopiperazines [58]. Similarly, the symbiotic microbes of *Dysidea* sp. consistently synthesize brominated diphenyl ethers [60]. Likewise, irrespective of the geographical region the antileukemic compound asperazine is produced by *Aspergillus niger* from two different *Hyrtios* species. Also, the antileukemic and antitumor compound roridin A is produced by *Myrothecium* sp. present in *Spongia* sp. of Hawaii and *Axinella* sp. of South China Sea [70,74,76,83].

**Figure 2:**
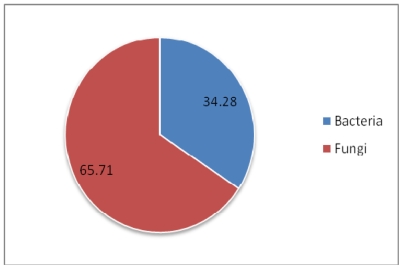
Percentage distribution of compounds produced by bacterial and fungal associates in sponges.

**Figure 3:**
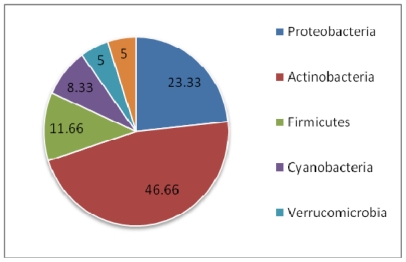
Percentage distribution of compounds produced by associated bacteria- phylum wise.

**Figure 4:**
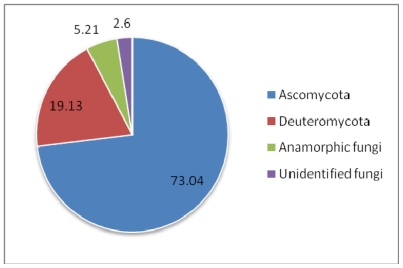
Percentage distribution of compounds produced by associated fungi-division wise.

Figures 2, 3 and 4 show the percentage distribution of clinically active compounds obtained from bacteria and fungi. Even though the number of bacterial isolates exhibiting clinical activities are more than fungi, many of the compounds produced by bacteria are not yet characterized. Figures 2 and 3 could be altered later once those compounds are characterized. Phylum Actinobacteria dominates in the production of therapeutic compounds followed by Proteobacteria. Bioactive potential of firmicutes and cyanobacteria is yet to be explored. Among fungi, Ascomycota is a predominant producer of bioactive molecules and Deuteromycota is also a potential group exhibiting bioactivity.

A wide range of chemical and functional diversity has been observed among bioactive compounds. Of the various chemical classes of compounds, polyketides, alkaloids, fatty acids, peptides and terpenes are the most abundant ones. Majority of them show antimicrobial, antitumor and anticancer properties. Bacterial and fungal associates in the order Dictyoceratida are found to synthesize antiangiogenic, anticancer, antiHIV, antitumor as well as antimicrobial compounds [15,40,57,59,61,62,64–66,69]. Another noticeable fact is the discovery of an actinobacterial strain (*Nocardiopsis dassonvillei* MAD08) from the sponge *Dendrilla nigra* of the family Darwinellidae from southwest coast of India (Table 3). This particular strain was able to produce compounds exhibiting antimicrobial, antioxidant, hypocholesterolemic, nematicidal, antiandrogenic, hemolytic, anti-inflammatory and anticancer properties. Of the various compounds produced by this strain, hexadecanoic acid- methyl ester, n-hexadecanoic acid, hexadecanoic acid-ethyl ester, 9-octadecenoic acid (Z)-methyl ester, oleic acid and (E)-9-octadecenoic acid-ethyl ester have been shown to be multifunctional [54]. Similarly the fungal strain *Gymnascella dankaliensis* OUPS-N134 from *Halichondria japonica* was very potent and produced 12 antileukemic compounds [78,102–109].

Some of the drugs available in the market, which were previously isolated from various terrestrial microbial genera were also detected in the marine counterparts associated with the sponges. A fungistatic drug, griseofulvin used for dermatophytoses has been isolated from various terrestrial and marine strains of *Penicillium*. This drug has also been reported from *Penicillium* symbiont of the Mediterranean sponge *Axinella verrucosa* [85,186]. Similarly, a well known immunosuppressive and antibiotic drug, mycophenolic acid which was produced by various strains of *Penicillium* including *Penicillium stoloniferum* and *Penicillium roqueforti* has also been isolated from *Penicillium brevicompactum* associated with the sponge *Petrosia ficiformis* [130,187,188]. Thus, the bioactive potential of the genus *Penicillium* either marine or terrestrial origin, free living or symbiotic makes it a worthy candidate for understanding the microbe-sponge association and harnessing the bioactive compounds.

## 4. Ecological and Cultural Aspects of Sponge Symbionts

To date, the primary target for marine bioprospecting has been tropical seas particularly coral reefs and other highly diverse ecosystems such as mangroves and seagrass because they host a high level of biodiversity and often face intense competition for space, leading to a chemical warfare among the sessile organisms. It was proven extremely difficult and in some cases impossible to provide sufficient quantity of these substances from invertebrates. The reason was due to the limited quantity of the compound, or still due to limited number of organisms producing the compound. Geographical, seasonal or sexual variations in the amount and nature of secondary metabolites could also be the other reasons for not consistently getting the required quantity of the compound. Marine invertebrates which are abundant in the Indo-Pacific regions, are rich in secondary metabolites and are becoming targets of continuing search for bioactive compounds [189]. The China Sea has become an important source of marine natural compounds since 2001 [190]. Among metazoans, the phylum Porifera contains the taxa which produce the highest diversity of secondary metabolites [191].

With some exceptions, sponge-associated microbial communities appear to be relatively stable with time and space [192]. With respect to temporal variability, the fluctuation of microbial communities in *Aplysina aerophobha* (an aquarium maintained specimen), *Geodia barrette* (Cultivated explant), temperate Australian sponges such as *Callyspongia* sp., *Stylinos* sp. and *Cymbastela concentrica* were detected to be low with no evidence of major seasonal changes [169,193,194]. In contrast to these studies, the bacterial community abundant in the North Sea sponge *Halichondria panacea* was found to vary considerably over a 10 month period [195]. Spatial variability could be ascribed to difference in microbiota within and among individuals which are separated by geographical barriers [194–196]. Marked differences were evident between the microbial communities inhabiting the outer (cortex) and inner (endosome) tissue in the Mediterranean sponge *Tethya aurantium* [197]. Contrary to this, Antarctic sponges such as *Homaxinella balfourensis*, *Kirkpatrickia varialosa*, *Latrunculia apicalis*, *Mycale acerata* and *Sphaerotylus antarcticus* collected from different sampling sites separated by 10 km were found to possess highly consistent bacterial communities. It highlights that site variability does not affect bacterial community composition in Antarctic sponges, but is highly consistent within a particular species [195]. Another study by Taylor *et al.* [198], showed that bacterial communities associated with temperate and tropical population of *Cymbastela concentrica* along the eastern Australian coast vary substantially.

Seasonal changes in the production of bioactive compounds by sponges are poorly understood. Seasonal fluctuations occurring in temperate seas impose significant alterations on the biology of the organism [199]. Seasonal changes, both qualitative and quantitative, have been observed in bioactive compound production in the sponge *Crambe crambe* from Mediterranean Sea. More importantly, high intra individual variability has also been observed. High toxicity in the producer organism during autumn may be a defense mechanism to counter increased growth of competing animal species at the end of summer. A decrease in toxicity in the months preceding April could be due to the reproductive rhythm. Energy diversion towards reproduction may explain the decrease in toxic metabolite production [200]. It was also found that non-polar fraction of the crude extract obtained from associated bacteria of the sponge *Ircinia ramosa* possessed strong antibacterial activity in summer. During winter season, activity was detected in the polar fraction and it was comparatively weaker than the observed activity in non-polar fraction during summer. This give insight in to the assumption that chemistry and production rate of metabolites from sponges or associated bacteria could be governed by environmental conditions [201]. More studies are being done to show that microbes are the real source of many of the bioactive compounds in sponges. Future efforts may throw more light on seasonal effects of bioactive compound production by these associates.

The occurrence of important metabolites within sponge-associated bacteria opens up the possibility of providing a continuous supply of the biologically active compounds by laboratory cultivation of the producer [202]. It would seem a logical step trying to isolate and cultivate putative bacterial producers outside invertebrate hosts in order to set up a sustainable and manageable source of pharmacologically active compounds. Even if microbial populations can be successfully separated from the hosts, the undefined metabolic factors of the host may render it difficult for the symbiont to grow *ex hospite* [82]. Many bacterial inhabitants in sponges appear to be highly selective with regard to culture media and conditions which probably reflect their evolutionary adaptation to the conditions provided by the host. Attempts to culture the theopaulamide producing bacteria from the sponge *Theonella swinhoei* have failed so far [203]. A notable exception is an anti-infective alkaloid manzamine A, which was successfully obtained from the culture of bacterium *Micromonospora* sp. of the deep-water Indonesian sponge *Acanthostrongylophora* sp. [13]. Another possibility is to grow the entire sponge and its microbial community in self-contained aquaculture systems for the economic, sustainable supply of important metabolites. The advantage of the latter strategy compared with growth of sponges in the wild or in open-water mariculture system is the possibility of better control of environmental conditions such as temperature, light, food supply and possibly precursors of important bioactive metabolites. In addition, aquaculture of sponges may provide less perturbation of the bacterium-host association over growth of bacterial ‘producers’ strains in pure culture which could be very important for maintaining production of compounds of interest [62].

It is hypothesized that antagonism, polyketide synthase genes and PLA2 are the key functional precursors of secondary metabolite synthesis and/or host defense of marine sponges. The study of metabolite-related genes of microorganisms associated with sponges may give insight into the origin of sponge-derived natural products. Polyketides, comprising a large and structural diverse family of bioactive natural products are one of the most important classes of marine natural compounds [190]. Polyketide synthase genes of host sponge and associated bacteria are predicted to be biosynthetic modules of polyketide analogues as well as phospholipases [51]. The PKS gene-based molecular approach can be applied to efficient screening of strains of pharmaceutical value and prediction of related compounds. This strategy has been employed to discover the efficiency of polyketide production in Firmicutes especially *Bacillus*, Actinobacteria and Proteobacteria isolated from sponges of the South China Sea [190]. Isolation and culture of symbiotic microorganisms as producers of secondary metabolites as well as transfer of symbiont biosynthetic genes into cultivable bacteria are subjects of ongoing research [149,204]. Even if compounds or compound groups appear exclusive for a particular taxon, they are not necessarily homologous and derived from a common ancestor and therefore do not necessarily reflect a genealogical relationship. They might originate from different precursors and biochemical pathways [203]. Rajdasa *et al*. [2], highlighted the repetitive PCR method as a powerful tool in estimating the richness of secondary metabolite producers among colonizers of sponge *Haliclona* sp. and this approach may be useful in studying the diversity of other sponge-associated microorganisms.

## 5. Conclusions

Sponge-microbial associations are found to be very specific in the production of particular bioactive compounds. However, the mutual mechanism between host and the microbial associate, in compound production is not well understood. The easiest and best way for commercial production of these compounds are either by culturing the host and/or the associated microbe under controlled conditions. But, the ability of the symbiont to produce the compound consistently for several generation in culture media has to be tested and standardized. Moreover, there is a need for quantifying the role of sponge ecology in orchestrating the production of specific compounds. Metagenomic approaches are also being increasingly used for targeting putative genes encoding potential metabolites in uncultured microbial biota. These approaches would help in delineating the contribution of either the host or microbial associate or both partners in the production of metabolites. A few compounds have been found to be produced both in terrestrial and marine ecosystems by different groups of host-symbiont association. This suggests the possibility of horizontal gene transfer through evolution. Discovery of potent microbial associates producing therapeutic compounds has opened up a new era in marine pharmacology. Understanding the optimum ecological conditions which drives the sustainable production of bioactive compounds from sponges and their microbial associates would help in formulating various production strategies. Adopting different cultivation strategies and metagenomic approaches would be the need of the hour in discovering new genes, enzymes and natural products and in enhancing the commercial production of marine drugs.

## Figures and Tables

**Figure 1:**
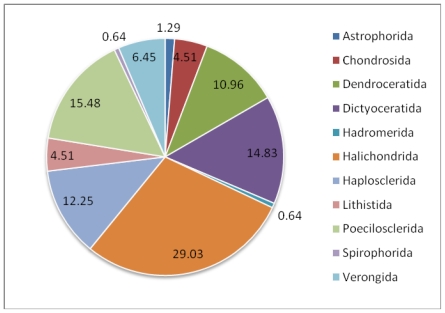
Percentage distribution of compounds produced by different orders of Demospongiae in association with microbes.

**Table 1:** Current status of species producing clinically active compounds in association with microbes.

Class: Demospongiae			
Order	Family	Species	Reference

Astrophorida	Ancorinidae	*Stelletta tenuis*	[23,42]

		*Jaspis aff. johnstoni*	[43–45]

Chondrosida	Chondrillidae	*Chondrosia reniformis*	[46–48]

Dendroceratida	Darwinellidae	*Dendrilla nigra*	[50–53]

Dictyoceratida	Dysideidae	*Lamellodysidea herbacea*	[40,57]
		*Dysidea* sp.	[59]
		*Dysidea avara*	[61,62]
	Irciniidae	*Ircinia fasciculata*	[33,64,65]
	Spongiidae	*Hyatella* sp.	[66]
		*Spongia* sp.	[69]
	Thorectidae	*Hyrtios altum*	[70–72]
		*Hyrtios* sp.	[45,73]
		*Hyrtios proteus*	[75]
		*Fascaplysinopsis reticulata*	[48,76]

Hadromerida	Spirastrellidae	*Spirastrella vagabunda*	[77,78]
	Suberitidae	*Suberites domuncula*	[15,79]

Halichondrida	Axinellidae	*Ptilocaulis trachys*	[81,82]
		*Axinella* sp. 1	[82]
		*Axinella* sp. 2	[84]
		*Axinella verrucosa*	[85–87]
		*Axinella damicornis*	[88]
		*Axinella* sp. 3	[89]
	Halichondriidae	*Halichondria okadai*	[78,93–97]
		*Halichondria panacea*	[100,101]
		*Halichondria japonica*	[79,102,104,109–113]
		*Acanthella acuta*	[116]
		*Hymeniacidon perlevis*	[43,44,117]

Haplosclerida	Callyspongiidae	*Callyspongia aerizusa*	[118]
		*Callyspongia vaginalis*	[119,120]
	Chalinidae	*Haliclona valliculata*	[121]
		*Haliclona simulans*	[25]
		*Haliclona* sp. 1	[123]
		*Haliclona* sp. 2	[2]
	Niphatidae	*Niphates olemda*	[124,125]
	Petrosiidae	*Petrosia ficiformis*	[129–131]
		*Xestospongia* sp.	[133]
		*Xestospongia exigua*	[79,124,134,135]
		*Acanthostrongylohpora* sp.	[82]
		*Petrosia* sp.	[139–141]

Lithistida	Neopeltidae	*Homophymia* sp.	[142]
	Theonellidae	*Theonella swinhoei*	[145–148,150]

Poecilosclerida	Acarnidae	*Zyzzya* sp.	[151,152]
	Isodictyidae	*Isodictya setifera*	[153]
	Raspailiidae	*Ectyoplasia ferox*	[154]
	Mycalidae	*Mycale plumose*	[159,160]
		*Mycale adhaerens*	[48,162]
	Myxillidae	*Myxilla incrustance*	[79,158]

Spirophorida	Tetillidae	*Craniella australiensis*	[163]

Verongida	Aplysinellidae	*Suberea clavata*	[167]
	Aplysinidae	*Aplysina aerophoba*	[170,174]
		*Aplysina cavernicola*	[41]
	Pseudoceratinidae	*Pseudoceratina purpurea*	[90]

**Class: Calcarea**			
	Leucettidae	*Leucetta microraphis*	[120]

**Table 2:** Clinically important bioactive compounds from sponge-microbe associations.

Class: Demospongiae					
**Order** Astrophorida **Family** Ancorinidae	**Sponge**	**Symbiont**	**Compound**	**Property**	**Reference**

	*Stelletta tenuis* (South China Sea)	*Alcaligenes faecalis* A72 (β-Proteobacteria)	Cyclo-(L-Pro-L-Phe)	Antimicrobial	[42]
	*Jaspis aff. johnstoni* (Indo-Pacific)	Hyphomycete fungus (Deuteromycota (fungus))	Chloriolin B	Antitumor	[43–45]

**Order** Chondrosida **Family** Chondrillidae	*Chondrosia reniformis* (Elba, Italy)	*Penicillium rugulosum* (Ascomycota (fungus))	Prugosene A1	Anti-infective	[46–48]
			Prugosene A2	Anti-infective	[46–48]
			Prugosene A3	Anti-infective	[46–48]
			Prugosene B1	Anti-infective	[46–48]
			Prugosene B2	Anti-infective	[46–48]
			Prugosene C1	Anti-infective	[46–48]
			Prugosene C2	Anti-infective	[46–48]

**Order** Dendroceratida **Family** Darwinellidae	*Dendrilla nigra* (Vizhinjam, India)	*Streptomyces dendra* sp. *nov.* MSI051 (Actinobacteria)	Unidentified compound	Antibacterial	[51]

	*Dendrilla nigra* (Kanyakumari, India)	*Streptomyces* sp. BLT7 (Actinobacteria)	Unidentified compound	Antibacterial	[52,53]

	*Dendrilla nigra* (South east coast, India)	*Nocardiopsis dassonvillei* MAD08 (Actinobacteria)	Acetic acid,-butyl-ester	Antimicrobial	[53]
			Ethanol, 2-(octyloxy)-	Antimicrobial	[53]
			Oxalic acid, allyl-nonyl ester	Antimicrobial	[54]
			2-Isopropyl-5-methyl-1-heptanol	Antimicrobial	[53]
			Butylated-hydroxytoluene	Antimicrobial	[53]
			Cyclohexane-carboxylic acid, hexyl ester	Antimicrobial	[53]
			Diethyl-phthalate	Antimicrobial	[53]
			Pentadecanal-	Antimicrobial	[53]
			1-Tridecanol	Antimicrobial	[53]
			9-Octadecenal	Antimicrobial	[53]
			Hexadecanoic acid, methyl-ester	Antioxidant, hypo-cholesterolemic, nematicide, antiandrogenic, hemolytic	[53]
			n-Hexadecanoic-acid	Antioxidant, hypo-cholesterolemic, nematicide, antiandrogenic, hemolytic	[53]
			Hexadecanoic-acid, ethyl ester	Antioxidant, hypo-cholesterolemic, nematicide, antiandrogenic, hemolytic	[53]
			9-Octadecenoic-acid-(Z)-, methyl-ester	Anti-inflammatory, antiandrogenic, cancer-preventive, dermatitigenic, hypo-cholesterolemic, anemiagenic	[53]
			Oleic Acid	Anti-inflammatory, antiandrogenic, cancer-preventive, dermatitigenic, hypo-cholesterolemic, anemiagenic	[53]
			(E)-9-Octadecenoic-acid ethyl ester	Anti-inflammatory, antiandrogenic, cancer-preventive, dermatitigenic, hypo-cholesterolemic, anemiagenic	[53]
			9-Octa-decenamide-(Z)-	Anti-inflammatory, antiandrogenic, cancer-preventive, dermatitigenic, hypo-cholesterolemic, anemiagenic	[53]

**Order** Dictyoceratida **Family** Dysideidae	*Lamellodysidea herbacea* (Great Barrier Reef, Australia)	*Oscillatoria spongeliae* (Cyanobacteria)	Dihydrodysamide C	Therapeutic (unknown action)	[57]
			Didechloro-dihydrodysamide C	Therapeutic (unknown action)	[57]

	*Dysidea* sp. (Eastern Samoa)	*Vibrio* sp. (γ-Proteobacteria)	Tetrabromo-diphenyl ethers	Cytotoxic, antibacterial	[59]

	*Lamellodysidea herbacea* (Republic of Palau)	*Oscillatoria spongeliae* (Cyanobacteria)	2-(2′,4′-dibromo-phenyl)-4,6-dibromophenol	Anibacterial	[40]

	*Dysidea avara* (Adriatic Sea)	Unidentified bacterium	2-methylthio-1,4-naphthoquinone	Antiangiogenic, antimicrobial	[61,62]

Irciniidae	*Ircinia fasciculata* (Mediterranean Sea)	*Penicillium chrysogenum* (Ascomycota (fungus))	Sorbicillactone A	Antileukemic, anti HIV	[64,65]

Spongiidae	*Hyatella* sp.	*Vibrio* sp. M22-1 (γ-Proteobacteria)	Andrimid	Antibiotic	[66]

	*Spongia* sp. (Hawaii)	*Myrothecium verrucaria* 973023 (Deuteromycota (fungus))	3-hydroxyroridin E	Antileukemic, antitumor	[69]
			13′-acetyl-trichoverrin B	Antileukemic, antitumor	[69]
			Roridin A	Antileukemic, antitumor	[69]
			Roridin L	Antileukemic, Antitumor	[69]
			Roridin M	Antileukemic, Antitumor	[69]
			Verrucarin M	Antileukemic, antitumor	[69]
			Verrucarin A	Antileukemic, antitumor	[69]
			Isororidin A	Antileukemic, antitumor	[69]
			Epiroridin E	Antileukemic, antitumor	[69]
			Trichoverrin A	Antileukemic, antitumor	[69]
			Trichoverrin B	Antileukemic, antitumor	[69]

Thorectidae	*Hyrtios altum* (Okinawa)	*Vibrio* sp. (γ-Proteobacteria)	Trisindoline	Antibiotic	[70–72]

	*Hyrtios* sp. (Caribbean Sea)	*Aspergillus niger* (Ascomycota (fungus))	Asperazine	Antileukemic, cytotoxic	[45,74,75]

	*Hyrtios proteus* (Dry Tortugas National Park, Florida )	*Aspergillus niger* (Ascomycota (fungus))	Asperazine	Antileukemic, cytotoxic	[76]
			Malformin C	Antitumor	[76]
	*Fascaplysinopsis reticulate* (Great Barrier Reef, Australia)	*Pseudo-alteromonas maricaloris* KMM 636T (γ-Proteobacteria)	Bromo-alterochromide A	Cytotoxic	[48,76]

			Bromo-alterochromide A	Cytotoxic	[48,76]

**Order** Hadromerida **Family** Spirastrellidae	*Spirastrella vagabunda* (Indonesia)	Unidentified fungus	14,15-secocurvularin	Antibiotic	[77,78]

Suberitidae	*Suberites domuncula* (Northern Adriatic Sea)	α-Proteobacterium MBIC3368 (isolate 1)	Unidentified compound	Antiangiogenic, antimicrobial, hemolytic, cytotoxic	[15,79]
		α-Proteobacterium MBIC3368 (isolate 2)	Unidentified compound	Antimicrobial, hemolytic	[15,80]
		*Idiomarina* sp. (γ-Proteobacteria)	Unidentified compound	Hemolytic	[15,80]
		*Pseudomonas* sp. (isolate 1) (γ-Proteobacteria)	Unidentified compound	Hemolytic, cytotoxic	[15,80]
		*Pseudomonas* sp. (isolate 2) (γ-Proteobacteria)	Unidentified compound	Antiangiogenic, antimicrobial, hemolytic, cytotoxic	[15,80]

**Order** Halichondrida **Family** Axinellidae	*Ptilocaulis trachys* (Enewetak Atoll, Marshall Island, Pacific Ocean)	*Lyngbya majuscula* (Cyanobacteria)	Majusculamide C	Antifungal	[81,82]

	*Axinella* sp. (South China Sea)	*Myrothecium* sp. JS9 (Deuteromycota (fungus))	Roridin A	Antifungal	[83]
			Roridin D	Antifungal	[83]

	*Axinella* sp. (Papaua New Guinea)	*Penicillium citrinum* (Ascomycota (fungus))	Isocyclocitrinol A	Antibacterial	[84]
			22-acetyl-isocyclocitrinol A	Antibacterial	[84]

	*Axinella verrucosa* (Mediterranean Sea)	*Penicillium* sp. (Ascomycota (fungus))	Oxaline	Anti-proliferative	[85]
			Griseofulvin	Antifungal	[85,86]
			Communesin B	Antileukemic	[85,87]
			Communesin C	Antileukemic	[85,87]
			Communesin D	Antileukemic	[85,87]

	*Axinella* sp. (Papua New Guinea)	*Acremonium* sp. (Ascomycota (fungus))	Efrapeptin E	Cytotoxic, antibacterial	[90]
			Efrapeptin F	Cytotoxic, antibacterial	[90]
			Efrapeptin Eα	Cytotoxic, antibacterial	[90]
			Efrapeptin G	Cytotoxic, antibacterial	[89]
			Efrapeptin H	Cytotoxic, antibacterial	[90]
			RHM1	Antibacterial	[89]

	*Axinella damicornis* (Mediterranean Sea)	*Aspergillus niger* (Ascomycota (fungus))	Bicoumanigrin	Anticancer, cytotoxic	[88]
			Aspernigrin B	Neuroprotective	[88]

Halichondriidae	*Halichondria okadai*	*Alteromonas* sp. (γ-Proteobacteria)	Alteramide A	Anticancer, cytotoxic	[93–95]

	*Halichondria okadai* (Japan)	*Trichoderma harzianum* OUPS-N115 (Ascomycota (fungus))	Trichodenone A	Antileukemic, cytotoxic	[78,96,97]
			Trichodenone B	Antileukemic, cytotoxic	[78,96,97]
			Trichodenone C	Antileukemic, cytotoxic	[78,96,97]

	*Halichondria okadai*	*Rubritalea squalenifasciens* HOact23^T^ (Verrucomicrobiae)	Dia-polycopenedioic acid xylosyl esters A	Antioxidant	[30,48,98]
			Dia-polycopenedioic acid xylosyl esters B	Antioxidant	[30,48,98]
			Dia-polycopenedioic acid xylosyl esters C	Antioxidant	[30,48,98]

	*Halichondria panacea*	Unidentified bacterium	Unidentified compound	Neuroactive	[100]

	*Halichondria panacea* (Adriatic coast, Croatia)	*Microbacterium* sp. (Actinobacteria)	1-O-acyl-3-[R-glucopyranosyl-(1–3)-(6-O-acyl-R-manno-pyranosyl)]-glycerol	Antitumor	[101]

	*Halichondria japonica* (Osaka Bay, Japan)	*Gymnascella dankaliensis* OUPS-N134 (Ascomycota (fungus))	Gymnostatin A	Antileukemic, cytotoxic	[78,102,103,105]
			Gymnostatin B	Antileukemic, cytotoxic	[78,102,103,105]
			Gymnostatin C	Antileukemic, cytotoxic	[78,102,103,105]
			Gymnostatin F	Antileukemic, cytotoxic	[106]
			Gymnostatin G	Antileukemic, cytotoxic	[106]
			Gymnostatin Q	Antileukemic, anti cancer, cytotoxic	[107]
			Gymnostatin R	Antileukemic, cytotoxic	[107]
			Gymnasterone A	Cytotoxic	[108,109]
			Gymnasterone B	Antileukemic, cytotoxic	[108,109]
			Gymnasterone C	Antileukemic, cytotoxic	[108]
			Gymnasterone D	Antileukemic, cytotoxic	[108]
			Dankastatin A	Antileukemic, cytotoxic	[107]
			Dankastatin B	Antileukemic, cytotoxic	[107]
			Dankasterone A	Antileukemic, anticancer, cytotoxic	[104]

	*Halichondria Japonica* (Japan)	*Phoma* sp. Q60596 (Ascomycota (fungus))	YM-202204	Antifungal	[110]

	*Halichondria Japonica*	*Bacillus cereus* QN03323 (Firmicutes)	YM-266183	Antibacterial	[111–113]
			YM-266184	Antibacterial	[111–113]

	*Acanthella acuta* (Mediterranean Sea)	*Bacillus pumilus* AAS3 (Firmicutes)	GG11	Antitumor	[116]

	*Hymeniacidon perlevis* (Nanji Island, China Sea)	*Pseudo-alteromonas piscicida* NJ6-3-1 (γ-Proteobacteria)	Norharman	Antimicrobial	[43,117]

	*Hymeniacidon perlevis* (Fujiazhuang coast, China)	*Fusarium oxysporum* DLFP2008005 (Ascomycota (fungus))	Unidentified compound	Antibacterial, antifungal	[44]

**Order** Haplosclerida **Family** Callyspongiidae	*Callyspongia aerizusa* (Indonesia)	*Cladosporium herbarum* (Deuteromycota (fungus))	Sumiki’s acid	Antibacterial	[118]
			Acetyl Sumiki’s acid	Antibacterial	[118]

	*Callyspongia vaginalis* (Caribbean Sea)	*Ulocladium botrylis* 193A4 (Ascomycota (fungus))	Ulocladol	Antimicrobial	[119,120]
			1-hydroxy-6-methyl-8-(hydroxylmethyl)-xanthone	Antifungal	[119,120]

Chalinidae	*Haliclona valliculata* (Elba, Italy)	*Emericella variecolor* (Ascomycota (fungus))	Evariquinone	Anti-proliferative	[122]

	*Haliclona simulans* (Ireland)	*Pseudo-alteromonas* sp. PA2 (γ-Proteobacteria)	Unidentified	Antimicrobial	[123]
		*Pseudo-alteromonas* sp. PA4 (γ-Proteobacteria)	Unidentified	Antimicrobial	[123]
		*Pseudo-alteromonas* sp. PA5 (γ-Proteobacteria)	Unidentified	Antimicrobial	[123]
		*Pseudo-alteromonas* sp. PA5 (γ-Proteobacteria)	Unidentified	Antimicrobial	[123]
		*Halomonas* sp. HM4 ( γ-Proteobacteria)	Unidentified	Antimicrobial	[123]
		*Psychrobacter* sp. PB1 (γ-Proteobacteria)	Unidentified	Antimicrobial	[123]
		*Marinobacter* sp. MB1 (γ-Proteobacteria)	Unidentified	Antimicrobial	[123]
		*Pseudovibrio* sp. PV1 (α-Proteobacteria)	Unidentified	Antimicrobial	[123]
		*Pseudovibrio* sp. PV2 ( α-Proteobacteria)	Unidentified	Antimicrobial	[123]
		*Pseudovibrio* sp. PV4 ( α-Proteobacteria)	Unidentified	Antimicrobial	[123]
		*Streptomyces* sp. SM1 (Actinobacteria)	Unidentified	Antimicrobial	[123]
		*Streptomyces* sp. SM2 (Actinobacteria)	Unidentified	Antimicrobial	[123]
		*Streptomyces* sp. SM3 (Actinobacteria)	Unidentified	Antimicrobial	[123]
		*Streptomyces* sp. SM4 (Actinobacteria)	Unidentified	Antimicrobial	[123]
		*Streptomyces* sp. SM5 (Actinobacteria)	Unidentified	Antimicrobial	[123]
		*Streptomyces* sp. SM6 (Actinobacteria)	Unidentified	Antimicrobial	[123]
		*Streptomyces* sp. SM7 (Actinobacteria)	Unidentified	Antimicrobial	[123]
		*Streptomyces* sp. SM8 (Actinobacteria)	Unidentified	Antimicrobial	[123]
		*Streptomyces* sp. SM9 (Actinobacteria)	Unidentified	Antimicrobial	[123]
		*Streptomyces* sp. SM10 (Actinobacteria)	Unidentified	Antimicrobial	[123]
		*Streptomyces* sp. SM11 (Actinobacteria)	Unidentified	Antimicrobial	[123]
		*Streptomyces* sp. SM12 (Actinobacteria)	Unidentified	Antimicrobial	[123]
		*Streptomyces* sp. SM14 (Actinobacteria)	Unidentified	Antimicrobial	[123]
		*Streptomyces* sp. SM16 (Actinobacteria)	Unidentified	Antimicrobial	[123]
		*Streptomyces* sp. SM17 (Actinobacteria)	Unidentified	Antimicrobial	[123]
		*Streptomyces* sp. SM18 (Actinobacteria)	Unidentified	Antimicrobial	[123]
		*Streptomyces* sp. SM19 (Actinobacteria)	Unidentified	Antimicrobial	[123]
		*Bacillus* sp. BC1 (Firmicutes)	Unidentified	Antimicrobial	[123]
		*Bacillus* sp. BC2 (Firmicutes)	Unidentified	Antimicrobial	[123]

	*Haliclona* sp. (Tomini Bay, North Sulawesi, Indonesia)	Unidentified fungus	Hirsutanol A	Antibiotic	[123]
			*ent*-gloeosteretriol	Antibiotic	[123]

	*Haliclona* sp.(North Java Sea, Indonesia)	Unidentified bacterium 1	Unidentified	Antibacterial	[2]
		Unidentified bacterium 2	Unidentified	Antibacterial	[2]
		Unidentified bacterium 3	Unidentified	Antibacterial	[2]
		Unidentified bacterium 4	Unidentified	Antibacterial	[2]
		Unidentified bacterium 5	Unidentified	Antibacterial	[2]

Niphatidae	*Niphates olemda* (Indonesia)	*Curvularia lunata* (Ascomycota (fungus))	Lunatin	Antibacterial	[124,125]
			Cytoskyrin A	Antibacterial	[124,125]

Petrosiidae	*Petrosia ficiformis* (Capo S. Andrea, Elba, Italy)	*Penicillium brevicompactum* (Ascomycota (fungus))	Mycophenolic acid	Immuno-suppressant	[128]
		*Aspergillus insuetus* (Ascomycota (fungus))	Terretonins E	Inhibit mammalian mitochondrial respiratory chain	[132]
			Terretonins F	Inhibit mammalian mitochondrial respiratory chain	[132]

	*Petrosia* sp. (Jeju Island, Korea)	*Aspergillus versicolor* (Ascomycota (fungus))	Decumbenone A	Melanin inhibitor	[139]
			Fellutamide C	Cytotoxic	[140]

	*Xestospongia* sp. (Off Noumea (New Caledonia, southwest Pacific))	*Micrococcus luteus* R-1588-10 (Actinobacteria)	2,4,4′-trichloro-2′-hydroxy-diphenylether (Triclosan)	Antimicrobial	[133]
			Acyl-1-(acyl-6′-mannobiosyl)-3-glycerol (Lutoside)	Antimicrobial	[133]

	*Xestospongia exigua* (Bali Sea, Indonesia)	*Penicillium cf. montanense* (Ascomycota (fungus))	Xestodecalactone B	Antifungal	[79,134]

	*Xestospongia exigua* (Indonesia)	*Aspergillus versicolor* (Ascomycota (fungus))	Aspergillitine	Antibacterial	[124,135]

	*Acantho-strongylophora* sp. (Indonesia)	*Micromonospora* sp. (Actinobacteria)	Manzamine A	Antitumor, antimalarial	[82,137, 138]

**Order** Lithistida **Family** Neopeltidae	*Homophymi*a sp. (Off Touho, New Caledonia)	*Pseudomonas* sp. 1537-E7 (γ-Proteobacteria)	2-undecyl-4-quinolone	Antimalarial Anti HIV	[142]
			2-undecen-1′-yl-4-quinolone	Cytotoxic	[142]
			2-nonyl-4-hydroxy-quinoline *N*-oxide	Antibacterial, cytotoxic	[142]

Theonellidae	*Theonella swinhoei* (Palau)	Unidentified bacterium	Swinholide A	Cytotoxic	[144,145]
		*Candidatus Entotheonella palauensis* (δ-Proteobacteria)	Theopalauamide	Antifungal	[26,144,146]

	*Theonella swinhoei* (Philippines)	*Entotheonella palauenis* (δ-Proteobacteria)	Theonegramide	Antifungal	[145,147]

	*Theonella swinhoei* (Hachijojima Island, Japan)	Uncultured bacterium	Onnamide A	Antitumor	[149,150]

**Order** Poecilosclerida **Family** Acarnidae	*Zyzzya* sp. (Fiji)	*Penicillium brocae* (Ascomycota (fungus))	Brocaenol A	Cytotoxic	[151,152]
			Brocaenol B	Cytotoxic	[151,152]
			Brocaenol C	Cytotoxic	[151,152]

Isodictyidae	*Isodictya setifera* (Hut Point and Danger Slopes, Ross Island, Antarctica)	*Pseudomonas aeruginosa* (γ-Proteobacteria)	Cyclo*-*(L-proline-L-methionine)	Antibacterial	[153]

Raspailiidae	*Ectyoplasia ferox* (Dominica, Carribean Island)	*Coniothyrium* sp. 193477 (Deuteromycota (fungus))	(3*S*)-(3′,5′-dihydroxyphenyl) butan-2-one	Antimicrobial	[154]
			2-(1′(*E*)-propenyl)-octa-4(*E*),6(*Z*)-diene-1,2-Diol	Antimicrobial	[154]
			(3*R*) 6-methoxymellein	Antimicrobial	[154]
			(3*R*)-6-methoxy-7-chloromellein	Antimicrobial	[154]
			Crypto-sporiopsinol	Antimicrobial	[154]
		*Phoma* sp. (Ascomycota (fungus))	Epoxyphomalin A	Antitumor	[155]
		*Spicellum roseum* 193H15 (Deuteromycota (fungus))	Trichodermol	Anticancer	[156,157]
			8-deoxytrichothecin	Anticancer	[156,157]

Mycalidae	*Mycale plumose* (Qingdao coast, China)	*Saccharopolyspora* sp. nov. (Actinobacteria)	Metacyclo-prodigiosin	Anticancer	[159]
			Undecyl-prodigiosin	Anticancer	[159]
		*Penicillium auratiogriseum* (Ascomycota (fungus))	(S)-2,4-dihydroxy-1-butyl(4-hydroxy)-benzoate	Antitumor	[160]
			Fructigenin A	Antitumor	[160]
			Aurantiomide B	Cytotoxic	[161]
			Aurantiomide C	Cytotoxic	[161]

	*Mycale adhaerens*	*Exophiala pisciphila* N110102 (Ascomycota (fungus))	Exophilin A	Antibacterial	[48,162]

Myxillidae	*Myxilla incrustance* (Helgoland, Germany)	*Microsphaeropsis* sp. H5-50 (Anamorphic fungus)	Microsphaeropsisin	Antifungal	[79,154]
			(*R*)-mellein	Antimicrobial	[154]
			(3*R*,4*S*)-hydroxymellein	Antimicrobial	[154]
			(3*R*,4*R*)-hydroxymellein	Antimicrobial	[154]
			4,8-dihydroxy-3,4-dihydro-2*H*-naphthalen-1-one	Antimicrobial	[154]

**Order** Spirophorida **Family** Tetillidae	*Craniella australiensis* (South China Sea)	*Streptomyces* sp. DA11 (Actinobacteria)	Chitinase	Antifungal	[163]

**Order** Verongida **Family** Aplysinellidae	*Suberea clavata* (Great Barrier Reef, Australia)	*Salinospora* sp. (Actinobacteria)	Rifamycin B	Antibiotic	[164,166]
			Rifamycin SV	Antibiotic	[164,166]

Aplysinidae	*Aplysina aerophoba* (Mediterranean coast, France)	*Bacillus subtilis* A184 (Firmicutes)	Surfactin, iturin and fengycin	Antifungal, antibacterial, hemolytic	[170]
		*Bacillus subtilis* A190 (Firmicutes)	Surfactin	Antifungal, hemolytic	[170]
		*Bacillus subtilis* A202 (Firmicutes)	Iturin	Antifungal, hemolytic	[170]
		*Bacillus pumilus* A586 (Firmicutes)	Pumilacidin containing β-hydroxy fatty-acid	Antibacterial	[170]

	*Aplysina aerophoba* (Mediterranean Sea)	*Microsphaeropsis* sp. (Anamorphic fungus)	10-Hydroxy-18-methoxyl-betaenone	Protein Kinase C ɛ inhibitor	[174]

	*Aplysina aerophoba* (Banyuls sur Mer)	*Bacillus* sp. SB8 (Firmicutes)	Unidentified compound	Antibacterial	[41]

		*Bacillus* sp. SB17 (Firmicutes)	Unidentified compound	Antibacterial	[41]
		*Micrococcus* sp. SB58 (Actinobacteria)	Unidentified compound	Antibacterial	[41]
		*Enterococcus* sp. SB91 (Firmicutes)	Unidentified compound	Antibacterial	[41]
		*Arthrobacter* sp. SB95 (Actinobacteria)	Unidentified compound	Antibacterial	[41]
		Unidentified bacteria SB122	Unidentified compound	Antibacterial	[41]
		Unidentified bacteria SB144	Unidentified compound	Antibacterial	[41]
		α-Proteobacteria SB6	Unidentified compound	Antibacterial	[41]
		α-Proteobacteria SB55	Unidentified compound	Antibacterial	[41]
		α-Proteobacteria SB63	Unidentified compound	Antibacterial	[41]
		α-Proteobacteria SB89	Unidentified compound	Antibacterial	[41]
		α-Proteobacteria SB156	Unidentified compound	Antibacterial	[41]
		α-Proteobacteria SB197	Unidentified compound	Antibacterial	[41]
		α-Proteobacteria SB202	Unidentified compound	Antibacterial	[41]
		α-Proteobacteria SB207	Unidentified compound	Antibacterial	[41]
		α-Proteobacteria SB214	Unidentified compound	Antibacterial	[41]
		*Vibrio halioticoli* SB177 (γ-Proteobacteria)	Unidentified compound	Antibacterial	[41]
		*Pseudo-alteromonas* sp. SB181 (γ-Proteobacteria)	Unidentified compound	Antibacterial	[41]
		*Pseudo-alteromonas* sp. SB182 (γ-Proteobacteria)	Unidentified compound	Antibacterial	[41]
		*Pseudo-alteromonas* sp. SB183 (γ-Proteobacteria)	Unidentified compound	Antibacterial	[41]
		*Pseudo-alteromonas* sp. SB185 (γ-Proteobacteria)	Unidentified compound	Antibacterial	[41]
		*Pseud-oalteromonas* sp. SB186 (γ-Proteobacteria)	Unidentified compound	Antibacterial	[41]
		*Pseudo-alteromonas* sp. SB192 (γ-Proteobacteria)	Unidentified compound	Antibacterial	[41]
		*Pseudo-alteromonas* sp. SB194 (γ-Proteobacteria)	Unidentified compound	Antibacterial	[41]
		*Pseudo-alteromonas* sp. SB200 (γ-Proteobacteria)	Unidentified compound	Antibacterial	[41]
		*Pseudo-alteromonas* sp. SB208 (γ-Proteobacteria)	Unidentified compound	Antibacterial	[41]
		*Pseudo-alteromonas* sp. SB213 (γ-Proteobacteria)	Unidentified compound	Antibacterial	[41]

Pseudo-ceratinidae	*Pseudoceratina purpurea* (Fiji)	*Metarrhizium* sp. 001103 (Ascomycota (fungus))	Destruxin A	Antitumor	[90]
			Destruxin B2	Antitumor	[90]
			Desmethyl B	Antitumor	[90]
			E chlorohydrin	Antitumor	[90]
			E2 chlorohydrin	Antitumor	[90]

**Class: Calcarea**					
**Order** Clathrinida **Family** Leucettidae	*Leucetta microraphis* (Great Barrier Reef, Australia)	Unidentified cyanobacteria	Leucamide A	Antitumor	[120]

**Unidentified sponges**					
Unidentified	Unidentified (Venezuela)	*Emericella variecolor* (Ascomycota (fungus))	Varitriol	Anticancer	[176]
			Varixanthone	Antimicrobial	[176]

	Unidentified	*Streptomyces* sp. *Ni-80* (Actinobacteria)	Urauchimycin A	Antibiotic	[177]
			Urauchimycin B	Antibiotic	[177]

	Unidentified (Harrington Point, Otago Harbor, New Zealand)	*Microascus longirostris* SF-73 (Ascomycota (fungus))	Cathestatin A	Cysteine protease inhibitor	[178]
			Cathestatin B	Cysteine protease inhibitor	[178]
			Cathestatin C	Cysteine protease inhibitor	[178]

	Unidentified (Pohnpei, The federated state of Micronesia)	*Aspergillus ostianus* 01F313 (Ascomycota (fungus))	8-chloro-9-hydroxy-8,9-deoxyasperlactone	Antibacterial	[179]
			9-chloro-8-hydroxy-8,9-deoxyasperlactone	Antibacterial	[179]
			9-chloro-8-hydroxy-8,9-deoxyaspyrone	Antibacterial	[179]
			Aspinonene	Antileukemic	[48,180]
			Dihydroaspyrone	Antileukemic	[48,180]
			Aspergillide A	Antileukemic	[48,181]
			Aspergillide B	Antileukemic	[48,181]
			Aspergillide C	Antileukemic	[48,181]
		*Cryptosphaeria eunomia* (Ascomycota (fungus))	Diaporthein A	Antibacterial	[48,182]
			Diaporthein B	Antibacterial	[48,182]

**Table 3:** Chemical diversity of therapeutics produced by sponge-microbe associations.

Category	Chemical diversity

Antiandrogenic	Fattyacid esters, fatty acids
Antiangiogenic	Quinone
Anticancer	Quinone, steroid, fatty acid esters, fatty acids, diketopiperazine, alkaloid, terpenes, terpenoids, trichoverroids, prodigiosin derivative
AntiHIV	Quinolone derivative
Anti-inflammatory	Fatty acid esters, fatty acid
Antimalarial	Alkaloid, quinolone derivative
Antimicrobial	Polyketide, glycopeptides, α-pyrone derivative, peptide, protein, antimycin, lipopeptides, polybrominated biphenyl ether, cyclic depsipeptide, terpenes, pentaketides, furan carboxylic acid, alkaloid, diketopiperazine, anthraquinone, chromones, steroid, lactone, quinolone derivative, trisindole derivative, macrolactam, ethers, phenol derivative
Antiinfective	Polyketides
Antioxidant	Fatty acid esters, fatty acid, carotenoic acid
Anti-respiratory	Terpenoids
Antitumor	Diglucosyl-glycerol, polyketides, alkaloids, cyclopeptides, glycoglycerolipid, benzoic acid derivative, terpenoids, terpenes, trichoverroids
Hemolytic	Fatty acid ester, fatty acids
Hypocholesterolemic	Fatty acid ester, fatty acids
Immunosupressant	Mycophenolic acid
Melanin inhibitor	Polyketide
Nematicide	Fatty acid ester, fatty acids
Neuroactive	Unknown
Neuroprotective	Dihydropyridine

**Table 4:** Microbial groups in various orders of sponges producing functionally diverse therapeutics.

Symbiont		Sponge order		Compound fuction
Bacteria	↔	Dendroceratida	→	Antiandrogenic
Bacteria	↔	Dictyoceratida, Hadromerida	→	Antiangiogenic
Bacteria	↔	Halichondrida, Dendroceratida, Poecilosclerida	→	Anticancer
Fungi	↔	Dictyoceratida, Halichondrida, Haplosclerida, Poecilosclerida		
Bacteria	↔	Lithistida	→	AntiHIV
Fungi	↔	Dictyoceratida		
Fungi	↔	Chondrosida	→	Anti-infective
Bacteria	↔	Dendroceratida	→	Ant-inflammatory
Bacteria	↔	Lithistida, Haplosclerida	→	Antimalarial
Bacteria	↔	Astrophorida, Dendroceratida Dictyoceratida, Hadromerida, Haplosclerida, Halichondrida, Lithistida, Poecilosclerida, Spirophorida, Verongida	→	Antimicrobial
Fungi	↔	Hadromerida, Halichondrida, Haplosclerida, Poecilosclerida		
Bacteria	↔	Dendroceratida, Halichondrida	→	Antioxidant
Fungi	↔	Haplosclerida	→	Anti-respiratory
Bacteria	↔	Clathrinida, Halichondrida, Haplosclerida, Lithistida	→	Antitumor
Fungi	↔	Astrophorida, Dictyoceratida, Poecilosclerida, Verongida		
Bacteria	↔	Hadromerida, Dendroceratida	→	Hemolytic
Bacteria	↔	Dendroceratida	→	Hypocholesterolemic
Fungi	↔	Haplosclerida	→	Immunosuppressant
Fungi	↔	Haplosclerida	→	Melanin inhibitor
Bacteria	↔	Dendroceratida	→	Nematicide
Bacteria	↔	Halichondrida	→	Neuroactive
Fungi	↔	Halichondrida	→	Neuroprotective

## References

[1] Hentschel U Hopke J Horn M Friedrich AB Wagner M Hacker J Moore BS Molecular evidence for a uniform microbial community in sponges from different oceans Appl Environ Microbiol 2002 68 4431 4440 12200297 10.1128/AEM.68.9.4431-4440.2002 PMC124103

[2] Radjasa OK Sabdono A Junaidi Zocchi E Richness of secondary metabolite producing marine bacteria associated with sponge *Haliclona sp* Int J Pharm 2007 3 275 279

[3] Fusetani N Matsunaga S Bioactive sponge peptides Chem Rev 1993 93 1793 1806

[4] Lee YK Lee JH Lee HK Microbial symbiosis in marine sponges J Microbiol 2001 39 254 264

[5] Fieseler L Horn M Wagner M Hentschel U Discovery of the novel candidate Phylum “*Poribacteria*” in marine sponges Appl Environ Microbiol 2004 70 3724 3732 15184179 10.1128/AEM.70.6.3724-3732.2004 PMC427773

[6] Belarbi EH Gomez AC Chisti Y Camacho FG Grima EM Producing drugs from marine sponges Biotechnol Adv 2003 21 585 598 14516872 10.1016/s0734-9750(03)00100-9

[7] Thakur NL Müller WEG Biotechnological potential of marine sponges Curr Sci 2004 86 1506 1512

[8] Jensen PR Fenical W Strategies for the discovery of secondary metabolites from marine bacteria: ecological perspectives Annu Rev Microbiol 1994 48 559 584 7826019 10.1146/annurev.mi.48.100194.003015

[9] Bernan VS Greenstein M Maise WM Marine microorganisms as a source of new natural products Adv Appl Microbiol 1997 43 57 89 9097412 10.1016/s0065-2164(08)70223-5

[10] Haygood MG Schmidt EW Davidson SK Faulkner DJ Microbial symbionts of marine invertebrates: opportunities for microbial biotechnology J Molec Microbiol Biotechnol 1999 1 33 43 10941782

[11] Osinga R Armstrong E Burgess JG Hoffmann F Reitner J Schumann-Kindel G Sponge microbe associations and their importance for sponge bioprocess engineering Hydrobiologia 2001 461 55 62

[12] Proksch P Edrada RA Ebel R Drugs from the seas: current status and microbiological imblications Appl Microbiol Biotechnol 2002 59 125 134 12111137 10.1007/s00253-002-1006-8

[13] Taylor MW Radax R Steger D Wagner M Sponge associated microorganisms: Evolution, ecology and biotechnological potential Microbiol Mol Biol Rev 2007 71 295 347 17554047 10.1128/MMBR.00040-06 PMC1899876

[14] Wang G Diversity and biotechnological potential of the sponge-associated microbial consortia J Ind Microbiol Biotechnol 2006 33 545 551 16761166 10.1007/s10295-006-0123-2

[15] Thakur AN Thakur NL Indap MM Pandit RA Datar VV Müller WEG Antiangiogenic, antimicrobial and cytotoxic potential of sponge-associated bacteria Mar Biotechnol 2005 7 245 252 15776311 10.1007/s10126-004-4085-y

[16] Gunasekera AS Sfanos KS Harmody DK Pomponi SA McCarthy PJ Lopez JV An enhanced database of the microorganisms associated with deeper water marine invertebrates Appl Microbiol Biotechnol 2004 66 373 376 15599519 10.1007/s00253-004-1763-7

[17] Kobayashi J Ishibashi M Bioactive metabolites of symbiotic marine microorganism Chem Rev 1993 93 1753 1769

[18] Ridley CP Faulkner DJ Haygood MG Investigation of *Oscillatoria spongeliae* dominated bacterial communities in four dictyoceratid sponges Appl Environ Microbiol 2005 71 7366 7375 16269779 10.1128/AEM.71.11.7366-7375.2005 PMC1287642

[19] Alvarez B Crisp MD Driver F Hooper JNA Soest RWMV Phylogenetic relationships of the family Axinellidae (Porifera: Demospongiae) using morphological and molecular data Zool Scripta 2000 29 169 198

[20] Bergmann W Burke DC Contributions to the study of marine products. XXXIX. The nucleosides of sponges. III. Spongothymidine and spongouridine J Org Chem 1955 20 1501 1507

[21] Wang G Diversity and biotechnological potential of the sponge-associated microbial consortia J Ind Microbiol Biotechnol 2006 33 545 551 16761166 10.1007/s10295-006-0123-2

[22] Kennedy J Baker P Piper C Cotter PD Walsh M Mooij MJ Bourke MB Rea MC O’Connor PM Ross RP Hill C O’Gara F Marchesi JR Dobson ADW Isolation and analysis of bacteria with antimicrobial activities from the marine sponge *Haliclona simulans* collected from Irish waters Mar Biotechnol 2009 11 384 396 18953608 10.1007/s10126-008-9154-1

[23] Li Z He L Miao X Cultivable bacterial community from South China Sea sponge as revealed by DGGE fingerprinting and 16S rDNA phylogenetic analysis Curr Microbiol 2007 55 465 472 17896134 10.1007/s00284-007-9035-2

[24] Head IM Saunders JR Pickup RW Microbial Evolution, Diversity, and Ecology: A Decade of Ribosomal RNA Analysis of Uncultivated Microorganisms Microb Ecol 1998 35 1 21 9459655 10.1007/s002489900056

[25] Juretschko S Timmermann G Schmid M Schleifer K Pommerening-Röser A Koops H Wagner M Combined Molecular and Conventional Analyses of Nitrifying Bacterium Diversity in Activated Sludge: *Nitrosococcus mobilis* and *Nitrospira*-Like Bacteria as Dominant Populations Appl Environ Microbiol 1998 64 3042 3051 9687471 10.1128/aem.64.8.3042-3051.1998 PMC106813

[26] Schmidt EW Obraztsova AY Davidson SK Faulkner DJ Haygood MG Identification of the antifungal peptide-containing symbiont of the marine sponge *Theonella swinhoei* as a novel δ-Proteobacterium *Candidatus Entotheonella palauensis* Mar Biol 2000 136 969 977

[27] Vacelet J Gallissian M Virus-like particles in cells of the sponge *Virongia cavernicola* (demospongiae, dictyoceratida) and accompanying tissue changes J Invertebr Pathol 1978 31 246 254

[28] Olson JB Mccarthy PJ Associated bacterial communities of two deep-water sponges Aquat Microb Ecol 2005 39 47 55

[29] Hill M Hill A Lopez N Harriott O Sponge-specific bacterial symbionts in the Caribbean sponge, Chondrilla nucula (Demospongiae, Chondrosida) Mar Biol 2006 148 1221 1230

[30] Kasai H Katsuta A Sekiguchi H Matsuda S Adachi K Shindo K Yoon J Yokota A Shizuri Y *Rubritalea squalenifaciens* sp. nov., a squalene-producing marine bacterium belonging to subdivision 1 of the phylum ‘*Verrucomicrobia*’ Int J Syst Evol Microbiol 2007 57 1630 1634 17625207 10.1099/ijs.0.65010-0

[31] Enticknap JJ Kelly M Peraud O Hill RT Characterization of a culturable alphaproteobacterial symbiont common to many marine sponges and evidence for vertical transmission *via* sponge larvae Appl Environ Microbiol 2006 72 3724 3732 16672523 10.1128/AEM.72.5.3724-3732.2006 PMC1472332

[32] Thiel V Imhoff JF Phylogenetic identification of bacteria with antimicrobial activities isolated from Mediterranean sponges Biomol Eng 2003 20 421 423 12919828 10.1016/s1389-0344(03)00069-8

[33] Radjasa OK Martens T Grossart H Brinkhoff T Sabdono A Simmon M Antagonistic activity of a marine bacterium *Pseudoalteromonas luteoviolacea* TAB4.2 associated with coral *Acropora sp* J Biol Sci 2007 7 239 246

[34] Zhang L An R Wang J Sun N Zhang S Hu J Kuai J Exploring novel bioactive compounds from marine microbes Curr Opin Microbiol 2005 8 276 281 15939350 10.1016/j.mib.2005.04.008

[35] Newman DJ Hill RT New drugs from marine microbes: the tide is turning J Ind Microbiol Biotechnol 2006 33 539 544 16598493 10.1007/s10295-006-0115-2

[36] Perry NB Blunt JW Munro MHG Mycalamide A, an antiviral compound from a New Zealand sponge of the genus *Mycale* J Am Chem Soc 1988 110 4850 4851

[37] Hood KA West LM Northcote PT Berridge MV Miller JH Induction of apoptosis by the marine sponge (*Mycale*) metabolites, mycalamide A and pateamine Apoptosis 2001 6 207 219 11388670 10.1023/a:1011340827558

[38] Laport MS Santos OCS Muricy G Marine sponges: Potential sources of new antimicrobial drugs Curr Pharmaceut Biotechnol 2009 10 86 105 10.2174/138920109787048625 19149592

[39] Unson MD Holland ND Faulkner DJ A brominated secondary metabolite synthesized by the cyanobacterial symbiont of a marine sponge and accumulation of the crystalline metabolite in the sponge tissue Mar Biol 1994 119 1 11

[40] Hentschel U Schmid M Wagner M Fieseler L Gernert C Hacker J Isolation and phylogenetic analyses of bacteria with antimicrobial activities from the Mediterranean sponges *Aplysina aerophoba* and *Aplysina cavernicula* FEMS Microbiol Ecol 2001 35 305 312 11311441 10.1111/j.1574-6941.2001.tb00816.x

[41] Zheng Z Zeng W Huang Y Yang Z Li J Cai H Su W Detection of antitumor and antimicrobial activities in marine organism associated actinomycetes isolated from the Taiwan Strait, China FEMS Microbiol Lett 2000 188 87 91 10867239 10.1111/j.1574-6968.2000.tb09173.x

[42] Li Z Advances in marine microbial symbionts in the China Sea and related pharmaceutical metabolites Mar Drugs 2009 7 113 129 19597576 10.3390/md7020113 PMC2707038

[43] Zhang Y Mu J Feng Y Kang Y Zhang J Gu P Wang Y Ma L Zhu Y Broad-spectrum antimicrobial epiphytic and endophytic fungi from marine organisms: Isolation, bioassay and taxonomy Mar Drugs 2009 7 97 112 19597575 10.3390/md7020097 PMC2707037

[44] Biabani MAF Laatsch H Advances in chemical studies on low-molecular weight metabolites of marine fungi J Prakt Chem 1998 340 589 607

[45] Cheng X Varoglu M Abrell L Crews P Lobkovsky E Clardy J Chloriolins A-C, chlorinated sesquiterpenes produced by fungal cultures separated from a *Jaspis* marine sponge J Org Chem 1994 59 6344 6348

[46] Sufrin JR Finckbeiner S Oliver CM Marine-Derived Metabolites of *S-*Adenosylmethionine as Templates for New Anti-Infectives Mar Drugs 2009 7 401 434 19841722 10.3390/md7030401 PMC2763108

[47] Lang G Wiese J Schmaljohann R Imhoff JF New pentaenes from the sponge-derived marine fungus *Penicillium rugulosum*: structure determination and biosynthetic studies Tetrahedron 2007 63 11844 11849

[48] Blunt JW Copp BR Hu W Munro MHG Northcote PT Prinsep MR Marine natural products Nat Prod Rep 2009 26 170 244 19177222 10.1039/b805113p

[49] Selvin J Gandhimathi R Seghal Kiran G Shanmugha Priya S Ravji TR Hema TA Culturable heterotrophic bacteria from the marine sponge *Dendrilla nigra*: Isolation and phylogenetic diversity of actinobacteria Helgol Mar Res 2009 63 239 247

[50] Selvin J Exploring the antagonistic producer *Streptomyces* MSI051: Implications of polyketide synthase gene type II and a ubiquitous defense enzyme phospholipase A2 in the host sponge *Dendrilla nigra* Curr Microbiol 2009 58 459 463 19130125 10.1007/s00284-008-9343-1

[51] Selvin J Lipton AP Biopotentials of secondary metabolites isolated from marine sponges Hydrobiologia 2004 513 231 238

[52] Selvin J Joseph S Asha KRT Manjusha WA Sangeetha VS Jayaseema DM Antony MC Vinitha AJD Antibacterial potential of antagonistic *Streptomyces* sp. isolated from marine sponge *Dendrilla nigra* FEMS Microbiol Ecol 2004 50 117 122 19712370 10.1016/j.femsec.2004.06.007

[53] Selvin J Shanmughapriya S Gandhimathi R Kiran GS Ravji TR Natarajaseenivasan K Hema TA Optimization and production of novel antimicrobial agents from sponge associated marine actinomycetes *Nocardiopsis dassonvillei* MAD08 Appl Microbiol Biotechnol 2009 83 435 445 19190903 10.1007/s00253-009-1878-y

[54] Hinde R Pironet F Borowitzka MA Isolation of *Oscillatoria spongeliae*, the filamentous cyanobacterial symbiont of the marine sponge *Dysidea herbacea* Mar Biol 1994 119 99 104

[55] Arillo A Bavestrello G Burlando B Sara M Metabolic integration between symbiotic cyanobacteria and sponges: a possible mechanism Mar Biol 1993 117 159 162

[56] Unson MD Faulkner DJ Cyanobacterial symbiont biosynthesis of chlorinated metabolites from *Dysidea herbacea* (Porifera) Cell Mol Life Sci 1993 49 349 353

[57] Flowers AE Garson MJ Webb RI Dumdei EJ Charan RD Cellular origin of chlorinated diketopiperazines in the dictyoceratid sponge *Dysidea herbacea* (Keller) Cell Tissue Res 1998 292 597 607 9582417 10.1007/s004410051089

[58] Besada P Mamedova L Thomas CJ Costanzi S Jacobson KA Design and synthesis of new bicyclic diketopiperazines as scaffolds for receptor probes of structurally diverse functionality Org Biomol Chem 2005 3 2016 2025 15889186 10.1039/b416349d PMC3476468

[59] Elyakov GB Kuznetsova T Mikhailov VV Maltsev II Voinov VG Fedoreyev SA 1991 Brominated diphenyl ethers from a marine bacterium associated with the sponge *Dysidea* sp Cell Mol Life Sci 1991 47 632 633

[60] Scheuermayer M Pimentel-Elardo S Fieseler L Grozdanov L Hentschel U Proksch P Müller WEG Microorganisms of sponges: Phylogenetic diversity and biotechnological potential Frontiers in Marine Biotechnology Horizon Bioscience Norfolk, UK 2006 289 312

[61] Thakur NL Müller WEG Sponge-bacteria association: A useful model to explore symbiosis in marine invertebrates Symbiosis 2005 39 109 116

[62] Müller WEG Thakur NL Ushijima H Thakur AN Krasko A Pennec G Indap MM Perovic-Ottstadt S Schröder HC Lang G Bringmann G Matrix-mediated canal formation in primmorphs from the sponge *Suberites domuncula* involves the expression of a CD36 receptor-ligand system J Cell Sci 2004 117 2579 2590 15159453 10.1242/jcs.01083

[63] Mohamed NM Rao V Hamann MT Kelly M Hill RT Monitoring bacterial diversity of the marine sponge *Ircinia strobilina* upon transfer to aquaculture Appl Environ Microbiol 2008 74 4133 4143 18469126 10.1128/AEM.00454-08 PMC2446523

[64] Bringmann G Lang G Muhlbacher J Schaumann K Steffens S Rytik PG Hentschel U Morschhauser J Müller WEG Sorbicillactone A: A structurally unprecedented bioactive novel-type alkaloid from a sponge-derived fungus Prog Mol Subcell Biol 2003 37 231 253 15825646 10.1007/978-3-642-55519-0_9

[65] Bringmann G Gulder TAM Lang G Schmitt S Stöhr R Wiese J Nagel K Imhoff JF Large–scale biotechnological production of the antileukemic marine natural product sorbicillactone A Mar Drugs 2007 5 23 30 18463724 10.3390/md502023 PMC2365691

[66] Oclarit JM Okada H Ohta S Kaminura K Yamaoka Y Iizuka T Miyashiro S Ikegami S Anti-bacillus substance in the marine sponge, *Hyatella* species, produced by an associated *Vibrio* species bacterium Microbios 1994 78 7 16 8022309

[67] Fredenhagen A Tamura SY Kenny PTM Komura H Naya Y Nakanishi K Nishiyama K Sugiura M Kita H Andrimid, a new peptide antibiotic produced by an intracellular bacterial symbiont isolated from a brown planthopper J Am Chem Soc 1987 109 4409 4411

[68] Needham J Kelly MT Ishige M Andersen RJ Andrimid and moiramides A-C, metabolites produced in culture by a marine isolate of the bacterium *Pseudomonas fluorescens*: Structure elucidation and biosynthesis J Org Chem 1994 59 2058 2063

[69] Amagata T Rath C Rigot JF Tarlov N Tenney K Valeriote FA Crews P Structures and cytotoxic properties of trichoverroids and their macrolide analogues produced by saltwater culture of *Myrothecium verrucaria* J Med Chem 2003 46 4342 4350 13678412 10.1021/jm030090t

[70] Kobayashi M Kitagawa I Bioactive substances isolated from marine sponge, a miniature conglomerate of various organisms Pure Appl Chem 1994 66 819 826

[71] Kobayashi M Aoki S Gato K Matsunami K Kurosu M Kitagawa I Marine natural products. XXXIV. Trisindoline, a new antibiotic indole trimer, produced by a bacterium of *Vibrio* sp. separated from the marine sponge *Hyrtios altum* Chem Pharm Bull 1994 42 2449 2451 7697760 10.1248/cpb.42.2449

[72] Braekman J Daloze D Chemical and biological aspects of sponge secondary metabolites Phytochem Rev 2004 3 275 283

[73] Varoglu M Corbett TH Valeriote FA Crews P Asperazine, a selective cytotoxic alkaloid from a sponge-derived culture of *Aspergillus niger* J Org Chem 1997 62 7078 7079 11671801 10.1021/jo970568z

[74] Govek SP Overman LE Total synthesis of asperazine J Am Chem Soc 2001 123 9468 9469 11562240 10.1016/j.tet.2007.05.127 PMC1987397

[75] Varoglu M Crews P Biosynthetically diverse compounds from a saltwater culture of sponge derived *Aspergillus niger* J Nat Prod 2000 63 41 43 10650076 10.1021/np9902892

[76] Speitling M Smetanina OF Kuznetsova TA Laatsch H Bromoalterochromides A and A′, unprecedented chromopeptides from a marine Pseudoalteromonas maricaloris strain KMM 636T J Antibiot 2007 60 36 42 17390587 10.1038/ja.2007.5

[77] Abrell LM Borgeson B Crews P A new polyketide, secocurvularin, from the salt water culture of a sponge derived fungus Tetrahedon Lett 1996 37 8983 8984

[78] Bugni TS Ireland CM Marine derived fungi: a chemically and biologically diverse group of microorganisms Nat Prod Rep 2004 21 143 163 15039840 10.1039/b301926h

[79] Thakur NL Hentschel U Krasko A Pabel CT ANR AC Müller WEG Antibacterial activity of the sponge *Suberites domuncula* and its primmorphs: potential basis for epibacterial chemical defense Aquat Microb Ecol 2003 31 77 83

[80] Webster NS Hill RT The culturable microbial community of the Great Barrier Reef sponge *Rhopaloeides odorabile* is dominated by an α-Proteobacterium Mar Biol 2001 138 843 851

[81] Williams DE Burgoyne DL Rettig SJ Andersen RJ Fathi-Afshar ZR Allen TM The isolation of Majusculamide C from the sponge *Ptilocaulis trachys* collected in Enewetak and determination of the absolute configuration of the 2-methyl-3-aminopentanoic acid residue J Nat Prod 1993 56 545 551

[82] Dunlap WC Battershill CN Liptrot CH Cobb RE Bourne DG Jaspars M Long PF Newman DJ Biomedicinals from the phytosymbionts of marine invertebrates: A molecular approach Methods 2007 42 358 376 17560324 10.1016/j.ymeth.2007.03.001

[83] Xie LW Jiang SM Zhu HHl Sun W Ouyang YC Dai SK Li X Potential inhibitors against *Sclerotinia sclerotiorum*, produced by the fungus *Myrothecium* sp. associated with the marine sponge *Axinella* sp Eur J Plant Pathol 2008 122 571 578

[84] Amagata T Amagata A Tenney K Valeriote FA Lobkovsky E Clardy J Crews P Unusual C25 steroids produced by a sponge-derived *Penicillium citrinum* Org Lett 2003 5 4393 4396 14602008 10.1021/ol0356800

[85] Koizumi Y Arai M Tomoda H Omura S Oxaline, a fungal alkaloid, arrests the cell cycle in M phase by inhibition of tubulin polymerization Biochim Biophys Acta 2004 1693 47 55 15276324 10.1016/j.bbamcr.2004.04.013

[86] Kolachana P Smith MT Induction of kinetochore-positive micronuclei in human lymphocytes by the anti-fungal drug griseofulvin Mutat Res 1994 322 151 159 7521514 10.1016/0165-1218(94)90001-9

[87] Jadulco R Edrada RA Ebel R Berg A Schaumann K Wray V Steube K Proksch P New communesin derivatives from the fungus *Penicillium* sp. derived from the Mediterranean sponge *Axinella verrucosa* J Nat Prod 2004 67 78 81 14738391 10.1021/np030271y

[88] Hiort J Maksimenka K Reichert M Perovic-Ottstadt S Lin WH Wray V Steube K Schaumann K Weber H Proksch P Ebel R Müller WEG Bringmann G New natural products from the sponge-derived fungus *Aspergillus niger* J Nat Prod 2004 67 1532 1543 15387655 10.1021/np030551d

[89] Boot CM Tenney K Valeriote FA Crews P Highly N-methylated linear peptides produced by an atypical sponge-derived *Acremonium sp* J Nat Prod 2006 69 83 92 16441074 10.1021/np0503653 PMC3972007

[90] Boot CM Amagata T Tenney K Compton JE Pietraszkiewicz H Valeriote FA Crews P Four classes of structurally unusual peptides from two marine-derived fungi: structures and bioactivities Tetrahedon 2007 63 9903 9914 10.1016/j.tet.2007.06.034 PMC2390835 18820723

[91] Erpenbeck D Breeuwer JAJ van der Velde HC van Soest RWM Unravelling host and symbiont phylogenies of halichondrid sponges (Demospongiae, Porifera) using mitochondrial marker Mar Biol 2002 141 377 386

[92] Miki W Otaki N Yokoyama A Izumida H Shimidzu N Okadaxanthin, a novel C50-carotenoid from a bacterium *Pseudomonas* sp. KK10206C associated with a marine sponge *Halichondria okadai* Experientia 1994 50 684 686

[93] Kelecom A Secondary metabolites from marine microorganisms An Acad Bras Cienc 2002 74 151 170 11960184 10.1590/s0001-37652002000100012

[94] Shigemori H Bae MA Yazawa K Sasaki T Kobayashi J Alteramide A, a new tetracyclic alkaloid from a bacterium *Alteromonas* sp. associated with the marine sponge *Halichondria okadai* J Org Chem 1992 57 4317 4320

[95] Bhalla TC Sharma M Sharma NN Satyanarayana T Chand S Microbial production of flavours and fragrances; fats and oils; dyes; bioplastics (PHAS); polysaccharides; pharmacologically active substances from marine microbes; anticancer agents and microbial transformation Applied Microbiology National Science Digital Library NISCAIR New Delhi, India 2008 7 1 34

[96] Amagata T Usami Y Minoura K Ito T Numata A Cytotoxic substances produced by a fungal strain from a sponge: physico-chemical properties and structures J Antibiot 1998 51 33 40 9531985 10.7164/antibiotics.51.33

[97] Usami Y Ikura T Amagata T Numata A First total syntheses and configurational assignments of cytotoxic trichodenones A–C Tetrahedron-Asymmetry 2000 11 3711 3725

[98] Shindo K Asagi E Sano A Hotta E Minemura N Mikami K Tamesada E Misawa N Maoka T Diapolycopenedioic Acid Xylosyl Esters A, B, and C, Novel Antioxidative Glyco-C30-carotenoic Acids Produced by a New Marine Bacterium Rubritalea squalenifaciens J Antibiot 2008 61 185 191 18503197 10.1038/ja.2008.28

[99] Althoff K Schutt C Steffen R Batel R Müller WEG Evidence for a symbiosis between bacteria of the genus *Rhodobacter* and the marine sponge *Halichondria panicea*: Harbor also for putatively toxic bacteria? Mar Biol 1998 130 529 536

[100] Perovic S Wichels A Schutt C Gerdts G Pahler S Steffen R Müller WEG Neuroactive compounds produced by bacteria from the marine sponge *Halichondria panicea*: activation of the neuronal NMDA receptor Environ Toxicol Pharmacol 1998 6 125 133 10.1016/s1382-6689(98)00028-3 21781889

[101] Wicke C Hners M Wray V Nimtz M Bilitewski U Lang S Production and structure elucidation of glycoglycerolipids from a marine sponge associated *Microbacterium* species J Nat Prod 2000 63 621 626 10843572 10.1021/np990313b

[102] Numata A Amagata T Minoura K lto T Gymnastatins, novel cytotoxic metabolites produced by a fungal strain from a sponge Tetrahedon Lett 1997 38 5675 5678

[103] Amagata T Doi M Ohta T Minoura K Numata A Absolute stereostructures of novel cytotoxic metabolites, gymnastatins A–E, from a *Gymnascella* species separated from a *Halichondria* sponge J Chem Soc Perkin Trans 1 1998 1 3585 3599

[104] Amagata T Doi M Tohgo M Minoura K Numata A Dankasterone, a new class of cytotoxic steroid produced by a *Gymnascella* species from a marine sponge Chem Commun 1999 1321 1322

[105] Mayer AMS Marine Pharmacology in 1998: Antitumor and Cytotoxic Compounds Pharmacologist 1999 41 159 164

[106] Amagata T Minoura K Numata A 2006. Gymnastatins F-H, Cytostatic Metabolites from the Sponge-Derived Fungus *Gymnascella dankaliensis* J Nat Prod 2006 69 1384 1388 17067147 10.1021/np0600189

[107] Amagata T Tanaka M Yamada T Minoura K Numata A Gymnastatins and Dankastatins, Growth Inhibitory Metabolites of a *Gymnascella* Species from a *Halichondria* Sponge J Nat Prod 2008 71 340 345 18284207 10.1021/np070529a

[108] Amagata T Tanaka M Yamada T Doi M Minoura K Ohishi H Yamori T Numata A Variation in Cytostatic Constituents of a Sponge-Derived *Gymnascella dankaliensis* by Manipulating the Carbon Source J Nat Prod 2007 70 1731 1740 17988094 10.1021/np070165m

[109] Amagata T Minoura K Numata A Gymnasterones, novel cytotoxic metabolites produced by a fungal strain from a sponge Tetrahedron Lett 1998 39 3773 3774

[110] Nagai K Kamigiri K Matsumoto H Kawano Y Yamaoka M Shimoi H Watanabe M Suzuki K YM-202204, a new antifungal antibiotic produced by marine fungus *Phoma sp* J Antibiot 2002 55 1036 1041 12617512 10.7164/antibiotics.55.1036

[111] Nagai K Kamigiri K Arao N Suzumura K Kawano Y Yamaoka M Zhang H Watanabe M Suzuki K YM-266183 and YM-266184, novel thiopeptide antibiotics produced by *Bacillus cereus* isolated from a marine sponge. I. Taxonomy, fermentation, isolation, physico-chemical properties and biological properties J Antibiot 2003 56 123 128 12715871 10.7164/antibiotics.56.123

[112] Suzumura K Yokoi T Funatsu M Nagai K Tanaka K Zhang H Suzuki K YM-266183 and YM-266184, novel thiopeptide antibiotics produced by *Bacillus cereus* isolated from a marine sponge II. Structure elucidation J Antibiot 2003 56 129 134 12715872 10.7164/antibiotics.56.129

[113] Laatsch H Proksch P Müller WEG Marine bacterial metabolite Frontiers in Marine Biotechnology Horizon Bioscience Norfolk, UK 2006 225 288

[114] Hildebrand M Waggoner LE Lim GE Sharp KH Ridley CP Haygood MG Approaches to identify, clone, and express symbiont bioactive metabolite genes Nat Prod Rep 2003 21 122 142 15039839 10.1039/b302336m

[115] El Sayed KA Dunbar DC Perry TL Wilkins SP Hamann MT Marine natural products as prototype insecticidal agents J Agric Food Chem 1997 45 2735 2739 10.1021/jf0207880 PMC4969014 12670165

[116] Ramm W Schatton W Wagner-Dobler I Wray V Nimtz M Tokuda H Enjyo F Nishino H Beil W Heckmann R Lurtz V Lang S Diglucosyl-glycerolipids from the marine sponge-associated *Bacillus pumilus* strain AAS3: their production, enzymatic modification and properties Appl Microbiol Biotechnol 2004 64 497 504 14593508 10.1007/s00253-003-1471-8

[117] Zheng L Chen H Han X Line W Yan X Antimicrobial screening and active compound isolation from marine bacterium NJ6-3-1 associated with the sponge *Hymeniacidon perleve* World J Microbiol Biotechnol 2005 21 201 206

[118] Jadulco R Proksch P Wray V Sudarsono Berg A Grafe U New macrolides and furan carboxylic acid derivative from the sponge derived fungus *Cladosporium herbarum* J Nat Prod 2001 64 527 530 11325242 10.1021/np000401s

[119] Höller U König GM Wright AD A new tyrosine kinase inhibitor from a marine isolate of *Ulocladium botrytis* and new metabolites from the marine fungi *Asteromyces cruciatus* and *Varicosporina ramulosa* Eur J Org Chem 1999 1999 2949 2955

[120] König GM Kehraus S Seibert SF Abdel-Lateff A Müller D Natural products from marine organisms and their associated microbes Chem Bio Chem 2005 7 229 238 10.1002/cbic.200500087 16247831

[121] Yu S Deng Z Proksch P Lin W Oculatol, oculatolide and A-nor sterols from the sponge *Haliclona oculata* J Nat Prod 2006 69 1330 1334 16989529 10.1021/np0600494

[122] Bringmann G Lang G Steffens S Gunther E Schaumann K Evariquinone, isoemericellin, and stromemycin from a sponge derived strain of the fungus *Emericella variecolor* Phytochemistry 2003 63 437 443 12770594 10.1016/s0031-9422(03)00189-4

[123] Wang G Abrell LM Avelar A Borgeson BM Crews P New hirsutane based sesquiterpenes from salt water cultures of a marine sponge derived fungus and the terrestrial fungus *Coriolus consors* Tetrahedron 1998 54 7335 7342

[124] Bhadury P Mohammad BT Wright PC The current status of natural products from marine fungi and their potential as anti-infective agents J Ind Microbiol Biotechnol 2006 33 325 337 16429315 10.1007/s10295-005-0070-3

[125] Jadulco R Brauers G Edrada RU Ebel R Wray V Sudarsono Proksch P New metabolites from sponge derived fungi *Curvularia lunata* and *Cladosporium herbarum* J Nat Prod 2002 65 730 733 12027752 10.1021/np010390i

[126] Kim JS Im KS Jung JH New bioactive polyacetylenes from the marine sponge *Petrosia* sp Tetrahedron 1998 54 3151 3158

[127] Lim YJ Park HS Im KS Lee CO Hong J Lee M Kim D Jung JH Additional cytotoxic polyacetylenes from the marine sponge *Petrosia* sp J Nat Prod 2001 64 46 53 11170665 10.1021/np000252d

[128] Vacelet J Donadey C Electron microscope study of the association between some sponges and bacteria J Exp Mar Bio Ecol 1977 30 301 314

[129] Chelossi E Milanese M Milano A Pronzato R Riccardi G Characterisation and antimicrobial activity of epibiotic bacteria from *Petrosia ficiformis* (Porifera, Demospongiae) J Exp Mar Biol Ecol 2004 309 21 33

[130] Bringmann G Lang G Steffens S Schaumann K Petrosifungins A and B, novel cyclodepsipeptides from a sponge-derived strain of *Penicillium brevicompactum* J Nat Prod 2004 67 311 315 15043401 10.1021/np034015x

[131] Lemmens-Gruber R Kamyar MR Dornetshuber R Cyclodepsipeptides - Potential drugs and lead compounds in the drug development process Curr Med Chem 2009 16 1122 1137 19275616 10.2174/092986709787581761

[132] López-Gresa MP Cabedo N González-Mas MC Ciavatta MA Avila C Primo J Terretonins E and F, Inhibitors of the Mitochondrial Respiratory Chain from the Marine-Derived Fungus *Aspergillus insuetus* J Nat Prod 2009 72 1348 1351 19719247 10.1021/np900085n

[133] Bultel-Poncé V Debitus C Berge J Cerceau C Guyot M Metabolites from the sponge-associated bacterium *Micrococcus luteus* J Mar Biotechnol 1998 6 233 236 9852617

[134] Edrada RA Heubes M Brauers G Wray V Berg A Grafe U Wohlfarth M Muhlbacher J Schaumann K Sudarsono S Bringmann G Proksch P Online analysis of xestodecalactones A-C, novel bioactive metabolites from the fungus *Penicillium cf. montanense* and their subsequent isolation from the sponge *Xestospongia exigua* J Nat Prod 2002 65 1598 1604 12444683 10.1021/np020085c

[135] Lin W Brauers G Ebel R Wray V Berg A Sudarsono Proksch P Novel chromone derivatives from the fungus *Aspergillus versicolor* isolated from the marine sponge *Xestospongia exigua* J Nat Prod 2002 66 57 61 12542346 10.1021/np020196b

[136] El Sayed KA Kelly M Kara UAK Ang KKH Katsuyama T Dumbar DC Khan AA Hamann MT New manzamine alkaloids with potent activity against infectious diseases J Am Chem Soc 2001 123 1804 1808 11456797 10.1021/ja002073o

[137] Sakai R Higa T Jefford CW Bernardinelli G Manzamine A, a novel antitumor alkaloid from a sponge J Am Chem Soc 1986 108 6404 6405

[138] Ang KKH Holmes MJ Higa T Hamann MT Kara UAK *In vivo* antimalarial activity of the beta-carboline alkaloid manzamine A Antimicrob Agents Chemother 2000 44 1645 1649 10817722 10.1128/aac.44.6.1645-1649.2000 PMC89926

[139] Lee YM Mansoor TA Hong J Lee C-O Bae KS Jung JH Polyketides from a Sponge-Derived Fungus, Aspergillus versicolor Nat Prod Sci 2007 13 90 96

[140] Lee YM Dang HT Hong J Lee C-O Bae KS Kim DK Jung JH A Cytotoxic Lipopeptide from the Sponge-Derived Fungus *Aspergillus versicolor* Bull Korean Chem Soc 2010 31 205 208

[141] Fujii Y Asahara M Ichinoe M Nakajima H Fungal melanin inhibitor and related compounds from *Penicillium decumbens* Phytochemistry 2002 60 703 708 12127587 10.1016/s0031-9422(02)00196-6

[142] Bultel-Poncé V Berge J Debitus C Nicolas J Guyot M Metabolites from the sponge associated bacterium *Pseudomonas* species Mar Biotechnol 1999 1 384 390 10489417 10.1007/pl00011792

[143] Capon RJ Ford J Lacey E Gill JH Heiland K Friedel T Phoriospongin A and B: Two new nematocidal depsipeptides from the Australian marine sponges *Phoriospongia* sp. and *Callyspongia bilamellata* J Nat Prod 2002 65 358 363 11908978 10.1021/np010329d

[144] Bewley CA Faulkner DJ Lithistid sponges: Star performers or hosts to the stars Angew Chem Int Ed 1998 37 2162 2178 10.1002/(SICI)1521-3773(19980904)37:16<2162::AID-ANIE2162>3.0.CO;2-2 29711453

[145] Bewley CA Holland ND Faulkner DJ Two classes of metabolites from *Theonella swinhoei* are localized in distinct populations of bacterial symbionts Experientia 1996 52 716 722 8698116 10.1007/BF01925581

[146] Schmidt EW Bewley CA Faulkner DJ Theopalauamide, a bicyclic glycopeptide from filamentous bacterial symbionts of the lithistid sponge *Theonella swinhoei* from Palau and Mozambique J Org Chem 1998 63 1254 1258

[147] Bewley CA Faulkner DJ Theonegramide, an antifungal glycopeptide from the Philippine lithistid sponge *Theonella swinhoei* J Org Chem 1994 59 4849 4852

[148] Piel J Metabolites from symbiotic bacteria Nat Prod Rep 2004 21 519 538 15282634 10.1039/b310175b

[149] Piel J Hui D Wen G Butzke D Platzer M Fusetani N Matsunaga S Antitumor polyketide biosynthesis by an uncultivated bacterial symbiont of the marine sponge *Theonella swinhoei* Proc Natl Acad Sci 2004 101 16222 16227 15520376 10.1073/pnas.0405976101 PMC528957

[150] Grozdanov L Hentschel U An environmental genomics perspective on the diversity and function of marine sponge-associated microbiota Curr Opinion Microbiol 2007 10 215 220 10.1016/j.mib.2007.05.012 17574904

[151] Bugni TS Bernan VS Greenstein M Janso JE Maiese WM Mayne CL Ireland CM Brocaenols A–C: Novel polyketides from a marine-derived *Penicillium brocae* J Org Chem 2003 68 2014 2017 12608826 10.1021/jo020597w

[152] Ebel R Proksch P Müller WEG Secondary metabolites from marine-derived fungi Frontiers in Marine Biotechnology Horizon Scientific Press Norwich, UK 2006 73 143

[153] Jayatilake GS Thornton MP Leonard AC Grimwade JE Baker BJ Metabolites from an Antarctic sponge associated bacterium *Pseudomonas aeruginosa* J Nat Prod 1996 59 293 296 8882433 10.1021/np960095b

[154] Höller U König GM Wright AD Three new metabolites from marine derived fungi of the genera *Coniothyrium* and *Microsphaeropsis* J Nat Prod 1999 62 114 118 9917295 10.1021/np980341e

[155] Mohamed IE Gross H Pontius A Kehraus S Krick A Kelter G Maier A Fiebig H König GM Epoxyphomalin A and B, Prenylated Polyketides with Potent Cytotoxicity from the Marine-Derived Fungus *Phoma* sp Org Lett 2009 11 5014 5017 19813715 10.1021/ol901996g

[156] Kralj A Gurgui M König GM van Echten-Deckert G Trichothecenes induce accumulation of glucosylceramide in neural cells by interfering with lactosylceramide synthase activity Toxicol Appl Pharmacol 2007 225 113 122 17889095 10.1016/j.taap.2007.08.005

[157] Chatterjee S Kolmakova A Lactosylceramide synthase: From molecular biochemistry to biological function Lipids (sphingolipid metabolizing enzymes 2004) Research Signpost Trivandrum, India 2004 33 41

[158] Kralj A Kehraus S Krick A van Echten-Deckert G König GM Two new depsipeptides from the marine fungus *Spicellum roseum* Planta Med 2007 73 366 371 17354168 10.1055/s-2007-967131

[159] Liu R Cui C Duan L Gu Q Zhu W Potent *in Vitro* anticancer activity of metacycloprodigiosin and undecylprodigiosin from a sponge-derived actinomycete *Saccharopolyspora* sp *nov* Arch Pharm Res 2005 28 1341 1344 16392666 10.1007/BF02977899

[160] Xin ZH Zhu WM Gu QQ Fang LD Cui CB A new cytotoxic compound from *Penicillium auratiogriseum*, symbiotic or epiphytic fungus of sponge *Mycale plumose* Chin Chem Lett 2005 16 1227 1229

[161] Xin ZH Fang Y Du L Zhu T Duan L Chen J Gu Q Zhu W Aurantiomides A C, quinazoline alkaloids from the sponge-derived fungus *Penicillium aurantiogriseum* SP0-19 J Nat Prod 2007 70 853 855 17455978 10.1021/np060516h

[162] Doshida J Hasegawa H Onuki H Shimidzu N Exophilin A, a new antibiotic from a marine microorganism *Exophiala pisciphila* J Antibiot 1996 49 1105 1109 8982339 10.7164/antibiotics.49.1105

[163] Han Y Yang B Zhang F Miao X Li Z Characterization of antifungal chitinase from marine *Streptomyces* sp. DA11 associated with south China sea sponge *Craniella australiensis* Mar Biotechnol 2009 11 132 140 18626709 10.1007/s10126-008-9126-5

[164] Kim TK Garson MJ Fuerst JA Marine actinomycetes related to the ‘*Salinospora*’ group from the Great Barrier Reef sponge *Pseudoceratina clavata* Environ Microbiol 2005 7 509 518 15816928 10.1111/j.1462-2920.2005.00716.x

[165] Mincer TJ Jensen PR Kauffman CA Fenical W Widespread and persistent populations of a major new marine Actinomycete taxon in ocean sediments Appl Environ Microbiol 2002 68 5005 5011 12324350 10.1128/AEM.68.10.5005-5011.2002 PMC126404

[166] Kim TK Hewavitharana AK Shaw PN Fuerst JA Discovery of a new source of rifamycin antibiotics in marine sponge Actinobacteria by phylogenetic prediction Appl Environ Microbiol 2006 72 2118 2125 16517661 10.1128/AEM.72.3.2118-2125.2006 PMC1393243

[167] Lafi FF Garson MJ Fuerst JA Culturable bacterial symbionts isolated from two distinct sponge species (*Pseudoceratina clavata* and *Rhabdastrella globostellata*) from the Great Barrier Reef of Australia Microb Ecol 2005 50 213 220 16215644 10.1007/s00248-004-0202-8

[168] Friedrich AB Merkert H Fendert T Hacker J Proksch P Hentschel U Microbial diversity in the marine sponge *Aplysina cavernicola* (formerly *Verongia cavernicola*) analyzed by fluorescence *in situ* hybridization (FISH) Mar Biol 1999 134 461 470

[169] Friedrich AB Fischer I Proksch P Hacker J Hentschel U Temporal variation of the microbial community associated with the Mediterranean sponge *Aplysina aerophoba* FEMS Microbiol Ecol 2001 38 105 113

[170] Pabel CT Vater J Wilde C Franke P Hofemeister J Adler B Bringmann G Hacker J Hentschel U Antimicrobial activities and Matrix-Assisted Laser Desorption/Ionization Mass Spectrometry of *Bacillus* Isolates from the marine sponge *Aplysina aerophoba* Mar Biotechnol 2003 5 424 434 14730425 10.1007/s10126-002-0088-8

[171] Maget-Dana R Peypoux F Iturins, a special class of pore-forming lipopeptides: biological and physicochemical properties Toxicology 1994 87 151 174 8160184 10.1016/0300-483x(94)90159-7

[172] Besson F Peypoux F Michel G Delcambe L Mode of action of iturin A, an antibiotic isolated from *Bacillus subtilis* on *Micrococcus luteus* Biochem Biophys Res Commun 1978 81 297 304 96818 10.1016/0006-291x(78)91532-2

[173] Klich MA Lax AR Bland JM Inhibition of some mycotoxigenic fungi by iturin A, a peptidolipid produced by *Bacillus subtilis* Mycopathologia 2004 116 77 80 1780001 10.1007/BF00436368

[174] Brauers G Edrada RA Ebel R Proksch P Wray V Berg A Grafe U Schachtele C Totzke F Finkenzeller G Marme D Kraus J Munchbach M Michel M Bringmann G Schaumann K Anthraquinones and betaenone derivatives from the sponge-associated fungus *Microsphaeropsis* species: Novel inhibitors of protein kinases J Nat Prod 2000 63 739 745 10869191 10.1021/np9905259

[175] Mackay HJ Twelves CJ Protein kinase C: a target for anticancer drugs Endocr Relat Cancer 2003 10 389 396 14503915 10.1677/erc.0.0100389

[176] Malmstroem J Christophersen C Barrero AF Oltra JE Justicia J Rosales A Bioactive metabolites from a marine-derived strain of the fungus Emericella Variecolor J Nat Prod 2002 65 364 367 11908979 10.1021/np0103214

[177] Imamura N Nishijima M Adachi K Sano H Novel antimycin antibiotics, urauchimycins A and B, produced by marine actinomycete J Antibiot 1993 46 241 246 8468237 10.7164/antibiotics.46.241

[178] Yu C Curtis JM Walter JA Wright JLC Ayer SW Kaleta J Querengesser L Fathi-Afshar ZR Potent inhibitors of cysteine proteases from the marine fungus *Microascus longirostris* J Antibiot 1996 49 395 397 8642005 10.7164/antibiotics.49.395

[179] Namikoshi M Negishi R Nagai H Dmitrenok A Kobayashi H Three new chlorine containing antibiotics from a marine-derived fungus *Aspergillus ostianus* collected in Pohnpei J Antibiot 2003 56 755 761 14632284 10.7164/antibiotics.56.755

[180] Kito K Ookura R Yoshida S Namikoshi M Ooi T Kusumi T Pentaketides Relating to Aspinonene and Dihydroaspyrone from a Marine-Derived Fungus *Aspergillus ostianus* J Nat Prod 2007 70 2022 2025 17994702 10.1021/np070301n

[181] Kito K Ookura R Yoshida S Namikoshi M Ooi T Kusumi T New Cytotoxic 14-Membered Macrolides from Marine-Derived Fungus *Aspergillus ostianus* Org Lett 2008 10 225 228 18078344 10.1021/ol702598q

[182] Yoshida S Kito K Ooi T Kanoh K Shizuri Y Kusumi T 2008. Four Pimarane Diterpenes from Marine Fungus: Chloroform Incorporated in Crystal Lattice for Absolute Configuration Analysis by X-Ray Chem Lett 2007 36 1386

[183] Dettrakul S Kittakoop P Isaka M Nopichai S Suyarnsestakorn C Tanticharoen M Thebtaranonth Y Antimycobacterial pimarane diterpenes from the Fungus *Diaporthe* sp Bioorg Med Chem Lett 2003 13 1253 1255 12657257 10.1016/s0960-894x(03)00111-2

[184] Höller U Wright AD Matthee GF König GM Draeger S Aust H Schulz B Fungi from marine sponges: Diversity, biological activity and secondary metabolites Mycol Res 2000 104 1354 1365

[185] Müller WEG Grebenjuk VA Pennec G Schröder H Brummer F Hentschel U Müller IM Breter H Sustainable production of bioactive compounds by sponges-cell culture and gene cluster approach: A review Mar Biotechnol 2004 6 105 117 15085406 10.1007/s10126-002-0098-6

[186] Petit KE Mondeguer F Roquebert MF Biard JF Pouches YF Detection of griseofulvin in a marine strain of *Penicillium waksmanii* by ion trap mass spectrometry J Microbiol Methods 2004 58 59 65 15177904 10.1016/j.mimet.2004.03.004

[187] Muth WL Nash CH III Biosynthesis of Mycophenolic acid: Purification and characterization of S-Adenosyl-L-Methionine: Demethylmycophenolic Acid O-Methyltransferase Antimicrob Agents Chemother 1975 8 321 327 241289 10.1128/aac.8.3.321 PMC429313

[188] Engel G Milczewski KE Prokopek D Teuber M Strain-specific synthesis of mycophenolic acid by *P. roqueforti* in blue-veined cheese Appl Environ Microbiol 1982 43 1034 1040 16346004 10.1128/aem.43.5.1034-1040.1982 PMC244182

[189] Sabdono A Radjasa OK Microbial symbionts in marine sponges: Marine natural product factory J Coast Dev 2008 11 57 61

[190] Zhang W Zhang F Li Z Miao X Meng Q Zhang X Investigation of bacteria with polyketide synthase genes and antimicrobial activity isolated from South China Sea sponges J Appl Microbiol 2009 107 567 575 19302490 10.1111/j.1365-2672.2009.04241.x

[191] Müller WEG Brummer F Batel R Müller IM Schröder HC Molecular biodiversity. Case study: Porifera (Sponges) Naturwissenschaften 2003 90 103 120 12649752 10.1007/s00114-003-0407-6

[192] Hentschel U Usher KM Taylor MW Marine sponges as microbial fermenters FEMS Microbiol Ecol 2006 55 167 177 16420625 10.1111/j.1574-6941.2005.00046.x

[193] Hoffmann F Rapp HT Reitner J Monitoring microbial community composition by Fluorescence *In situ* Hybridization during cultivation of the marine cold-water sponge *Geodia barretti* Mar Biotechnol 2006 8 373 379 16758369 10.1007/s10126-006-5152-3

[194] Taylor MW Schupp PJ Dahllof I Kjelleberg S Steinberg PD Host specificity in marine sponge-associated bacteria, and potential implications for marine microbial diversity Environ Microbiol 2004 6 121 130 14756877 10.1046/j.1462-2920.2003.00545.x

[195] Webster NS Negri AP Munro MMHG Battershill N Diverse microbial communities inhabit Antarctic sponges Environ Microbiol 2004 6 288 300 14871212 10.1111/j.1462-2920.2004.00570.x

[196] Wichels A Wurtz S Dopke H Schutt C Gerdts G Bacterial diversity in the breadcrumb sponge *Halichondria panacea* (Pallas) FEMS Microbiol Ecol 2006 56 102 118 16542409 10.1111/j.1574-6941.2006.00067.x

[197] Thiel V Neulinger SC Staufenberger T Schmaljohann R Imhoff JF Spatial distribution of sponge-associated bacteria in the Mediterranean sponge *Tethya aurantium* FEMS Microbiol Ecol 2006 59 47 63 17059482 10.1111/j.1574-6941.2006.00217.x

[198] Taylor MW Schupp PJ Nys R Kjelleberg S Steinberg PD Biogeography of bacteria associated with the marine sponge *Cymbastela concentrica* Environ Microbiol 2005 7 419 433 15683402 10.1111/j.1462-2920.2004.00711.x

[199] Giese AC Pearse JS Giese AC Pearse JS Introduction: general principles Reproduction of Marine Invertebrates. Vol I. Acoelomate and Pseudocoelomate Metazoans Academic Press New York, USA 1974 1 49

[200] Turon X Becerro MA Uriz MJ Seasonal patterns of toxicity in benthic invertebrates: The encrusting sponge Crambe crambe (Pecilosclerida) Oikos 1996 75 33 46

[201] Thakur NL Anil AC Antibacterial activity of the sponge *Ircinia ramosa*: Importance of its surface-associatedbacteria J Chem Ecol 2000 26 57 71

[202] Imhoff JF Stohr R Sponge-associated bacteria: general overview and special aspects of bacteria associated with *Halichondria panicea* Prog Mol Subcell Biol 2003 37 35 57 15825639 10.1007/978-3-642-55519-0_2

[203] Erpenbeck D van Soest RWM Status and perspective of sponge chemosystematics Mar Biotechnol 2006 9 2 19 16817029 10.1007/s10126-005-6109-7

[204] Proksch P Edrada-Ebel R Ebel R Drugs from the Sea-Opportunities and obstacles Mar Drugs 2003 1 5 17

